# Impact of cooperative or competitive dynamics between the yeast *Saccharomyces cerevisiae* and lactobacilli on the immune response of the host

**DOI:** 10.3389/fimmu.2024.1399842

**Published:** 2024-10-10

**Authors:** Stefano Nenciarini, Damariz Rivero, Alessia Ciccione, Roberta Amoriello, Benedetta Cerasuolo, Marco Pallecchi, Gian Luca Bartolucci, Clara Ballerini, Duccio Cavalieri

**Affiliations:** ^1^ Department of Biology, University of Florence, Firenze, Italy; ^2^ Department of Experimental and Clinical Medicine, University of Florence, Florence, Italy; ^3^ Department of Neurosciences, Psychology, Drug Research and Child Health (NEUROFARBA), University of Florence, Florence, Italy; ^4^ Interuniversity Consortium for Biotechnologies, Trieste, Italy

**Keywords:** yeasts, lactobacilli, fermented food, host immune system modulation, *Saccharomyces cerevisiae*, microbial ecology, short-chain fatty acids, trained immunity

## Abstract

Fungi and bacteria can be found coexisting in a wide variety of environments. The combination of their physical and molecular interactions can result in a broad range of outcomes for each partner, from competition to cooperative relationships. Most of these interactions can also be found in the human gastrointestinal tract. The gut microbiota is essential for humans, helping the assimilation of food components as well as the prevention of pathogen invasions through host immune system modulation and the production of beneficial metabolites such as short-chain fatty acids (SCFAs). Several factors, including changes in diet habits due to the progressive Westernization of the lifestyle, are linked to the onset of dysbiosis statuses that impair the correct balance of the gut environment. It is therefore crucial to explore the interactions between commensal and diet-derived microorganisms and their influence on host health. Investigating these interactions through co-cultures between human- and fermented food-derived lactobacilli and yeasts led us to understand how the strains’ growth yield and their metabolic products rely on the nature and concentration of the species involved, producing either cooperative or competitive dynamics. Moreover, single cultures of yeasts and lactobacilli proved to be ideal candidates for developing immune-enhancing products, given their ability to induce trained immunity in blood-derived human monocytes *in vitro*. Conversely, co-cultures as well as mixtures of yeasts and lactobacilli have been shown to induce an anti-inflammatory response on the same immune cells in terms of cytokine profiles and activation surface markers, opening new possibilities in the design of probiotic and dietary therapies.

## 1 Introduction

Bacterial-fungal communities exist in virtually all habitats (1, 2), engaging in a variety of interactions within and between species, from symbiosis to competition and predation (3). The human body is an ecological habitat of special importance since numerous populations of bacteria and fungi, that compose the human microbiota together with archaea and viruses, regulate many aspects of human health (4–6). Fungi and bacteria interact in different modes throughout the human body environment: they can directly bind through physical interaction, release, and uptake chemical molecules, proliferate in mixed biofilms, and compete (7). For example, numerous studies on *C. albicans* describe mutualistic interactions with streptococci and competition with lactobacilli (8). On the other hand, *S. cerevisiae* is shown to exert inhibitory effects against *E. coli* (9) and enhance bacterial exopolysaccharide (EPS) production in *Lacticaseibacillus rhamnosus* (10). The interactions between yeasts and lactobacilli as members of the microbiota are not limited to humans, as confirmed by the evidence that *S. cerevisiae* strains reduce potentially pathogenic bacterial genera in the guts of wasps (11).

Humans have benefited from a life-long coexistence with bacterial-fungal communities for millennia, to produce food, antibiotics, and secondary metabolites for pharmacological and biotechnological purposes (12). Fermented foods, such as yogurt, kefir, and kombucha, known to host rich microbial communities, are traditionally part of diets around the world (13). The symbiotic relationship that exists within these microbial communities are not completely understood. An important study by Ponomarova and colleagues (14) demonstrated the role of specific *S. cerevisiae*-produced amino acids in promoting the growth of *Lactiplantibacillus plantarum*, *Lactobacillus plantarum* and *Lactococcus lactis in vitro* and in kefir.

Among the multiple factors that are known to shape both the bacterial and fungal components of the microbiota (15, 16), numerous studies demonstrate diet as a key factor (17–20). Rates of chronic inflammation statuses and non-communicable chronic diseases (NCCDs) are on the rise due to changes in diets, e.g. the so-called “Westernization”, a lifestyle condition also characterized by an unbalanced diet in terms of fat-fiber ratio (21, 22), which causes a strong reduction in microbiota diversity (23). On the contrary, fermented foods are the dietary basis for many populations with traditional lifestyles, enriching their microbiota with probiotic microbes with anti-inflammatory properties (24). Several studies support these findings by comparing microbiotas of traditional and industrialized lifestyles (25–28). Experimental diet interventions show significant changes in microbiota composition and immune status (29–31), confirming that the composition and functions of the human gut microbiota strongly influence the overall health status of an individual.

Dysbiosis statuses may impair the resistance to microbial colonization as well as host immune responses and cause the insurgence of several diseases under those with predisposed conditions. In the last decade, scientific research has increasingly explored the links between the microbiota and human health. Evidence has emerged that the composition of the entire community of microbial inhabitants, and not just one or two dominant species, influences a balanced immune response (24, 32). Aberrant immune responses to the gut microbiota, caused by dysbiosis, favor the onset of chronic inflammation and lead to inflammatory bowel diseases (IBD), i.e. ulcerative colitis and Crohn’s disease (33–36). Several studies connect dysbiosis with colorectal cancer (37); metabolic diseases such as obesity, type 2 diabetes, and food intolerances (38); neurological disorders through the microbiota-gut-brain (MGB) axis (39, 40); autoimmune and allergic diseases (41–44). In exchange for a favorable colonizing environment, a balanced gut microbiota carries out several beneficial functions for the host. It plays a fundamental role in the synthesis of vitamins and nutrients, as well as in the inhibition of pathogen invasion, by competing for intestinal ecological niches (45) and through the production of metabolites, such as short-chain fatty acids (SCFAs), which reflects in a reduction of virulence gene expression and growth rates of pathogens (46). Under healthy conditions, SCFAs are absorbed by intestinal epithelial cells, leading to the expression of antimicrobial peptides and the maintenance of epithelial integrity (47, 48), and exerting positive effects on the immune system cells in terms of inflammation and gut homeostasis (49).

Human Intestinal Epithelial Cells (IECs) and immune cells recognize microbe-associated molecular patterns (MAMPs) through pattern-recognition receptors (PRRs) and then discriminate between those harmless and pathogenic (50, 51). Effective immune response requires the direct action of innate immunity cells and the production of cytokines for adaptive immunity activation (52). Modulation of the host immune system exerted by the gut microbiota also relies on a so-called trained immunity, defined as a long-term functional modification of innate immune cells that leads to a greater response in case of a second unrelated immune challenge (53). A recent review of trained immunity during mucosal diseases highlights the potential for clinical treatment and emphasizes the importance of microbiota composition in modulating immunity (54).

Present research largely focuses on host-microbiota dynamics and their consequences for health, but the interactions between members of the microbiota are still poorly investigated, especially between beneficial microbes (7). Health status relies on all the microbiota interactions, including those between diet-derived microorganisms (55–57). Here, we performed an explorative investigation of the relationships within co-cultures of *S. cerevisiae* and *Lactobacillus* spp. isolated from different sources, including fermented milk (similar to kefir) previously collected by our group from the Yaghnob Valley in Tajikistan (58), a commercial probiotic, and the stool of a Crohn’s disease patient. Our results indicated that co-culture growth yield, trained immunity potential in humans *in vitro*, and SCFA production strongly depend on the natural sources of microorganisms.

## 2 Materials and methods

### 2.1 Selection of microbial strains

The bacterial strain of *Lactiplantibacillus plantarum* (B1) was isolated with the manufacturer’s consent from a commercial probiotic product that contained mixes of other lactobacilli. The *Lactobacillus delbrueckii* bacterial strain (TJA9) and *Saccharomyces cerevisiae* yeast strain (CL4) were isolated from a fermented goat milk beverage produced in the Yaghnob Valley in Tajikistan (58). The yeast strain *Saccharomyces cerevisiae* (YH1) was isolated from human fecal samples of a pediatric patient with Crohn’s disease (59, 60).

### 2.2 Culture assays

Given the high nutritional requirements of lactobacilli, which include adequate amino acids, vitamins, carbohydrates, and nucleotides (61, 62), the co-cultures were carried out in in De Man Rogosa Sharpe selective liquid medium (MRS) (Oxoid) + 0.05% cysteine HCl (63). Pre-cultures of lactobacilli were incubated overnight at 37°C in anaerobic conditions in MRS medium + 0.05% cystein HCl, whereas pre-cultures of yeasts were incubated overnight at 30°C in aerobic conditions in Yeast Peptone Dextrose (YPD) medium. Each strain pre-culture was diluted in fresh medium at a concentration of 2 x 10^6^ cells/ml for yeast and 2 x 10^6^ cells/ml or 2 x 10^7^ cells/ml for bacteria, with a yeast:bacteria ratio of 1:1 or 1:10. The inocula were incubated for 24 hours at 37°C in a shake at 200 r.p.m. The choice of temperature was due to the overall aim of the work, i.e. investigating how yeast and lactobacilli grow together in human-related conditions like the gut environment. To ensure that our yeast species could survive and reproduce at such temperature, we measured their growth after 24 hours at 30°C and 37°C. Although, as expected, 30° resulted in a more optimal condition, yeast cells could also grow at 37°C. We also assessed 37°C as optimal for the growth of the co-cultures. A representative result of the growth assays is available in **Supplementary Material** (**Supplementary Figure S2**). Several experiments were set up with the goals of studying the growth yield of different yeast strains of *Saccharomyces cerevisiae* and Lactobacillus in co-cultures and investigating the possible interactions that may occur between them. Microbial growth in experimental co-cultures were compared to those in single cultures. The growth of the strains was measured by Bürker chamber counts at different time points, and each experiment was set up in triplicate. The viability of the strains (both in mono-cultures and in co-cultures) was demonstrated by conducting colony counts on agar plates in three different experiments (three technical replicates for each experiment). The statistical test applied to assess differences in viability is the Wilcoxon-Mann-Whitney test (**Supplementary Table S1**).

### 2.3 SCFA’s analyses

For each GC-MS analysis, single and co-cultures were kept for 24 hours at 37°C in CDM_35, a chemically defined medium already validated for the co-cultures of lactobacilli and yeasts (14). Throughout the experiment, 2 ml of culture supernatants were collected at separate time points every hour for 8 hours in a row and again at completion. The cell growth was monitored by counting at the Bürker chamber. All conditions were set up in triplicate, and then the samples were stored at -80°C.

#### 2.3.1 Chemicals

Methanol and tert-butyl methyl ether (Chromasolv grade), sodium bicarbonate, sodium chloride and hydrochloric acid (Reagent grade), [2H5]Propionic, [2H7]iso-Butyric and [2H9]iso-Valeric (used as internal standards or ISTDs), acetic, propionic, butyric, iso-butyric, valeric, iso-valeric, 2-Methylbutyric, hexanoic, heptanoic, octanoic, and nonanoic acids (analytical standards grade) were purchased by Sigma-Aldrich (Milan, Italy). MilliQ water 18 MΩ cm was obtained from Millipore’s Simplicity system (Milan - Italy).

#### 2.3.2 GC-MS method

The qualitative and quantitative evaluation of fatty acids (FAs) was performed using the Agilent gas chromatography-mass spectrometry (GC-MS) system composed of a 5971 single quadrupole mass spectrometer, a 5890 gas-chromatograph, and a 7673 autosampler, through our previously described GC-MS method (64). The FAs were extracted as follows: an aliquot of 1.5 ml of medium culture sample was added to 10 μl of ISTD mixture, 0.5 ml of tert-butyl methyl ether (MTBE), and 100 μl of 6 M HCl, 0.5 M NaCl solution in 2 ml centrifuge tube. Afterward, each tube was stirred in a vortex for 3 minutes, centrifuged at 10,000 rpm, and finally the solvent layer was transferred into an autosampler vial and analyzed. The FAs in the samples were analyzed as free acid form using an Agilent J&W DB-FFAP column 30 m in length, 0.25 mm internal diameter, and 0.25 m of film thickness by using the oven temperatures’ program, as follows: initial temperature of 50°C for 1 min, then it was increased to 150°C at 30°C/min, finally grow up to 250°C at 20°C/min was held for 6.67 min. A 1 µl aliquot of extracted sample was injected in splitless mode (splitless time 1 min) at 250°C, while the transfer line temperature was 280°C. The used carrier gas was helium and its flow rate was maintained at 1 mL/min for the whole run time. The MS acquisition was carried out in single ion monitoring (SIM) by applying a proper dwell time (20 ms for each ion monitored) to guarantee an acquisition frequency of 4 cycle/s. The quantitative determination of FAs in each sample was carried out by the ratio between the area abundance of the analytes and the area abundance of the respective labeled internal standard (isotopic dilution method). The value of this ratio was named Peak Area Ratio (PAR) and it was used as the abundance of each analyte in the quantitative evaluation. The ionic FAs’ signals and the reference internal standards used for the quantitation of each FAs were reported in **Supplementary Table S2**.

#### 2.3.3 Data analysis

Before analyzing the FAs concentrations, negative control (fresh medium) values were subtracted from each sample. Samples were analyzed with GraphPad Prism version 9.5.0 for Windows. The performed statistical analysis were Linear regression and a Repeated Measures two-way ANOVA with the Geisser-Greenhouse correction. Time points were compared with Tukey’s multiple comparisons test, with individual variances computed for each comparison. Figures and statistics for CL4-B1 and YH1-B1 setups are available in **Supplementary Material** (**Supplementary Tables S3**-**S14**, **Supplementary Figures S3**, **S4**).

### 2.4 Immunological assays

All work with human study participants was approved by the Ethical Committees of the Azienda Ospedaliera Universitaria (AOU) Careggi (Ref. n. 87/10) and AOU Meyer Children’s Hospital (Ref. n. 103/2021), Florence, Italy. The research was carried out according to the principles set out in the Declaration of Helsinki 1964 and all subsequent revisions. Buffy coats were collected from fifteen anonymous healthy donors at the Transfusion Unit at Careggi University Hospital in Florence, Italy. The utilization of donor material, not destined to diagnostic standard procedures and registered with a traceable numeric code, was authorized by the Careggi Transfusion Unit.

#### 2.4.1 Immunomagnetic separation of monocytes

Differential centrifugation with Lympholyte^®^-H Cell Separation Media (EuroClone) at 2000 rpm for 20 minutes at room temperature separated peripheral blood mononuclear cells (PBMCs) from the remaining blood cell components. The ring of mononuclear cells at the plasma/lymphocyte interface was collected and washed twice with DPBS (Dulbecco’s Phosphate Buffered Saline, EuroClone) before centrifuging for 5 minutes at 2000 and 1200 rpm. The cells were resuspended in a sterile saline solution containing DPBS, 1% FBS (fetal bovine serum), and 2 mM EDTA (Buffer MACS). The cells were resuspended and centrifuged at 1200 rpm for 5 minutes. 100 μl of CD14 MicroBeads Human (Miltenyi Biotec) were added to the remaining pellet and incubated at 4°C for 15 minutes. Following the addition of 10 mL of MACS Buffer, CD14+ monocytes were isolated by positive magnetic separation using LS columns and the MidiMACSTM Separator immunomagnetic separator, according to the manufacturer’s protocol (Miltenyi Biotec). A volume of 5 ml MACS Buffer was added to the column to recover CD14+ cells. After elution, the cell suspension was centrifuged at 1200 rpm for 5 minutes, and the cell pellet was washed and resuspended in complete RPMI medium supplemented with 10% FBS (100X), 1% sodium pyruvate, 1X nonessential amino acids (100X), 1X Penicillin-Streptomycin (500X), and 20mM L-glutamine. Using the 0.22 m Vacuum Filter System, the entire RPMI culture medium was sterilized by vacuum filtration (EuroClone).

#### 2.4.2 Preparation of stimuli

*In vitro* experiments of trained immunity induction were carried out using the method described by Rizzetto and colleagues (59). Monocytes (10^6^ cells/ml) from fifteen healthy donors were first exposed to low concentrations (10^4^ cells/ml) of *S. cerevisiae*, Lactobacillus, or the combination *S. cerevisiae* + Lactobacillus in a 96- well flat bottom plate for 24 hours in a CO_2_ incubator at 37°C before being washed to remove all stimuli and incubated for 5 days preserving the same conditions. The cells were subsequently re-stimulated with pure Lipopolysaccharide (LPS, 10 ng/ml) and incubated with the second stimulus for 24 hours.

#### 2.4.3 Quantification of cytokines

The concentration of cytokines IL-6 and TNF-α was determined by ELISA immunoassay, employing the ELISA MAX™ Deluxe Set Human IL-6 and ELISA MAX™ Deluxe Set Human TNF-α (BioLegend), according to the protocol provided by the manufacturer. The inflammatory cytokine panel was measured simultaneously using the MILLIPLEX system (Merck Millipore) to determine whether co-cultures or mixtures induced more production of anti-inflammatory cytokines, such as IL-10, than pro-inflammatory cytokines, such as IL-6 and TNF.

#### 2.4.4 Phenotypic and functional characterization of monocytes

Monocyte surface markers were studied by cytofluorometry using the CyFlow Space 6-color (Sysmex Partec). The anti-human monoclonal antibodies used (Invitrogen) specifically recognized the following antigens (the fluorochrome tags are given in brackets): CD11b (FITC), CD14 (PE), CD80 (FITC), CD86 (APC), HLA-DR (PERCP). Data were acquired with the Sysmex Partec software FloMax.

#### 2.4.5 Data analysis

For the statistical analysis of immunological data, GraphPad Prism 9.5.0 software and the programming environment R 4.3.1 (65) were used. The monocyte markers analysis figure was graphically generated by the ggplot2 package (66). Results were expressed as means ± SEM and the performed statistical tests were one-way ANOVA followed by Tukey’s multiple comparison test. Statistical significance was for p values < 0.05.

## 3 Results

### 3.1 Microbial growth yield in co-culture

To study whether the interactions between yeast and Lactobacillus modify their growth, 24-hour co-cultures have been made between a bacterial and a yeast organism within the four selected strains. Although *S. cerevisiae* and lactobacilli are known to proliferate when grown together, we tested the viability of the strains by co-culturing YH1 and B1. We chose to test these two strains since they come from different biological matrices, while CL4 and TJA9 have been isolated from the same matrix, so we expected them to thrive in co-cultures. As expected, there were no statistically significant differences in the viability of both bacteria and yeasts between mono-cultures and co-cultures (**Supplementary Figure S1**).

#### 3.1.1 *Lactiplantibacillus plantarum’s growth yield* in co-culture depends on cellular concentration

**Figure 1:**
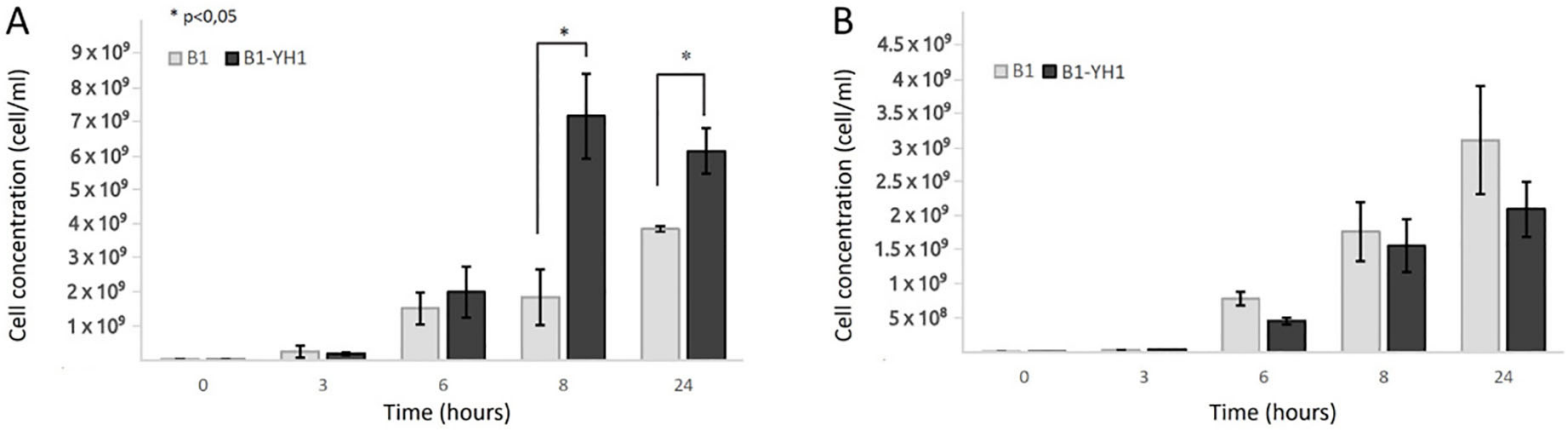
Counts over time of *L. plantarum* B1 in single culture and co-culture with *S. cerevisiae* YH1 in MRS medium at 37°C for 24 hours in triplicates, with a yeast-bacteria concentration ratio of 1:10 **(A)** or 1:1 **(B)**. Cellular concentrations were determined by cell counting at the Bürker chamber. Statistical significance was assessed by T-test, * for p < 0.05.

As depicted in **Figure 1**, in co-cultures of *S. cerevisiae* YH1 (human-derived) and *L. plantarum* B1 (commercial probiotic), the bacteria improved their growth yield after 8 hours of co-culture with the yeast, when the cell concentration ratio was 1:10 (**Figure 1A**). No significant differences were observed in the growth yield of the bacteria when the concentration ratio was 1:1 (**Figure 1B**). No change in the growth yield of YH1 in co-culture with B1 was observed (data not shown).

#### 3.1.2 *Lactobacillus delbrueckii’s* growth yield is not enhanced in co-culture with *S. cerevisiae*

**Figure 2:**
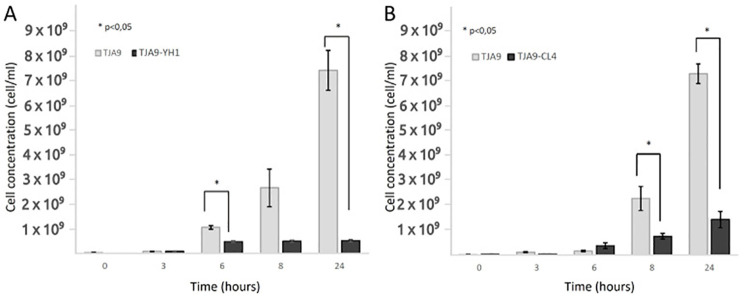
Counts over time of *L. delbrueckii* TJA9 in single culture and co-culture with *S. cerevisiae* strains YH1 **(A)** and CL4 **(B)** in MRS medium at 37°C for 24 hours in triplicates. Cellular concentrations were determined by cell counting in the Bürker chamber. Statistical significance was assessed by T-test, * for p < 0.05.

The increase in the bacterial growth yield shown in **Figure 1** led us to wonder whether other lactobacilli experienced the same effect. **Figure 2** showed that the L. delbrueckii TJA9 strain did not benefit from the co-culture with the yeast. On the contrary, its growth yield significantly decreased in co-culture (**Figure 2A**). The same result was observed using the CL4 yeast strain in co-culture, even if it was isolated from the same biological matrix as TJA9 (**Figure 2B**).

#### 3.1.3 *S. cerevisiae* growth yield increases in co-culture with *L. delbrueckii*

**Figure 3:**
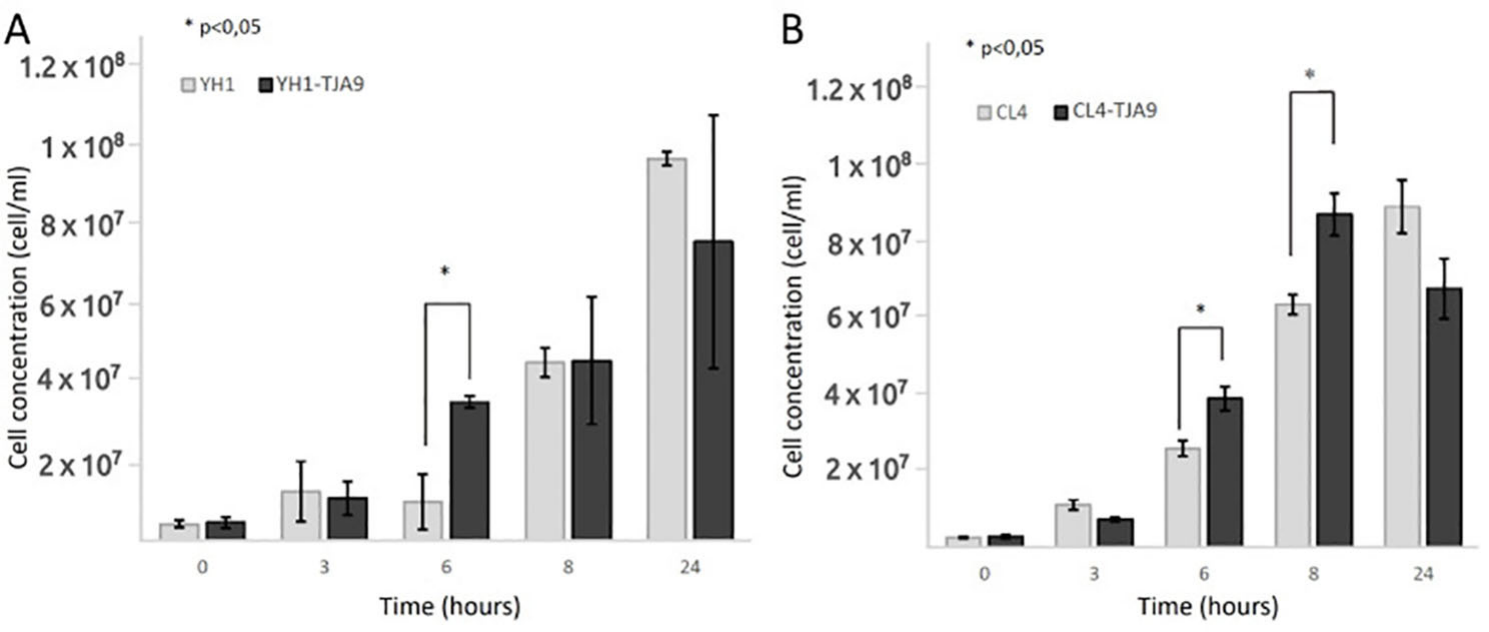
Counts over time of *S. cerevisiae* strains YH1 **(A)** and CL4 **(B)** in single culture and co-culture with *L. delbrueckii* TJA9 in MRS medium at 37°C for 24 hours in triplicates, with a yeast-bacteria concentration ratio of 1:10. Cellular concentrations were determined by cell counting at the Bürker chamber. Statistical significance was assessed by T-test, * for p < 0.05.

Given the growth yield reduction of TJA9 with both yeast strains, we checked if it was related to an increase in yeast growth yield under the same conditions. In **Figure 3** we showed that both strains of *S. cerevisiae* showed better growth in co-culture with TJA9 than in the single culture, even if this effect seems to reduce after 8 hours (**Figure 3**).

### 3.2 Mass spectrometry analysis of short-chain fatty acids

To evaluate if the co-culture between yeast and lactobacillus increases SCFAs production compared to the single cultures, gas-chromatography mass-spectrometry (GC-MS) analyses were performed. Yeast and lactobacilli were grown in a chemically defined medium, which allowed precise quantification of SCFAs produced by the microorganisms.

**Figure 4:**
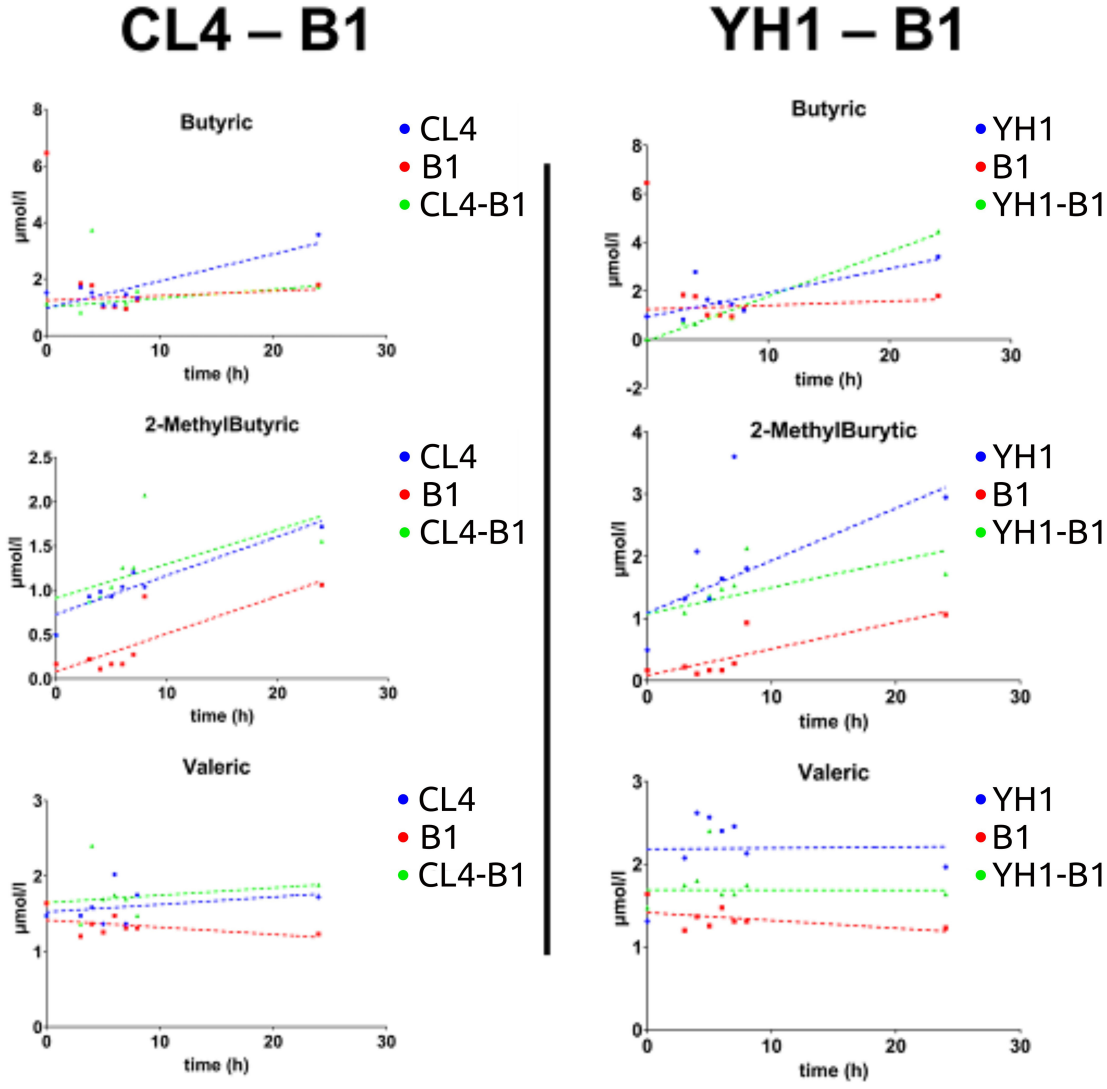
SCFAs (Butyric acid, 2-MethylButyric acid, and Valeric acid) production in chemically defined medium represented as time-scaled points and relative linear regression models (dotted lines). The SCFAs production is plotted as concentration (on the y-axis) in 8 different time points (on the x-axis) for the three studied conditions. Blue stands for yeast single cultures, red stands for bacterial single cultures, and green stands for co-cultures. Each value is the mean of three different biological replicates. Statistical significance for each experiment is shown in Supplementary material (**Supplementary Tables S3**, **S6**, **S7**, **S10**, **S12**, **S13**).

On average, SCFAs production followed the same pattern between all the co-cultures studied, showing that the values for yeast single cultures were significantly higher than those for bacterial cultures and co-cultures. Three exceptions were found: 2-MethylButyric acid and Valeric acid for the CL4-B1 setup, and Butyric acid for the YH1-B1 setup (**Figure 4**). Among these, the values of 2-MethylButyric acid for the CL4-B1 setup and Butyric acid for the YH1-B1 setup were significantly higher in the co-cultures than in the yeast cultures in at least one time point (**Supplementary Tables S3**-**S14**, **Supplementary Figures S3**, **S4**).

### 3.3 Immune assays

Multiple immune assays were performed to assess the inflammatory and immune-training potential of yeasts and lactobacilli both in single and co-cultures. The tests consisted of an *in vitro* first stimulation of human monocytes with the microorganisms, and subsequent stimulation with LPS after five days, followed by cytokine production levels assessment and immunophenotype analysis (the flow cytometry gating strategy is displayed in **Supplementary Figure S5**). To investigate whether the effect was due to the mere presence of the two organisms or the co-culture dynamics, a condition made of a mixture of yeast and lactobacilli was added as control. Before other immune assays, the first test was the assessment of monocytes’ viability at day 6 after 24 hours of treatment with yeast or bacteria single cultures, co-cultures, and mixtures. The assessment was performed through a specific kit that discriminates alive cells from apoptotic and necrotic ones. The results (**Supplementary Figure S6**) showed that in almost all conditions the monocytes’ viability was above 60% of the total cells. The decrease in viability can be attributed to a physiological death rate of human blood cells cultivated *in vitro* for 6 days.

#### 3.3.1 Yeasts and lactobacilli alone elicit trained immunity responses

**Figure 5:**
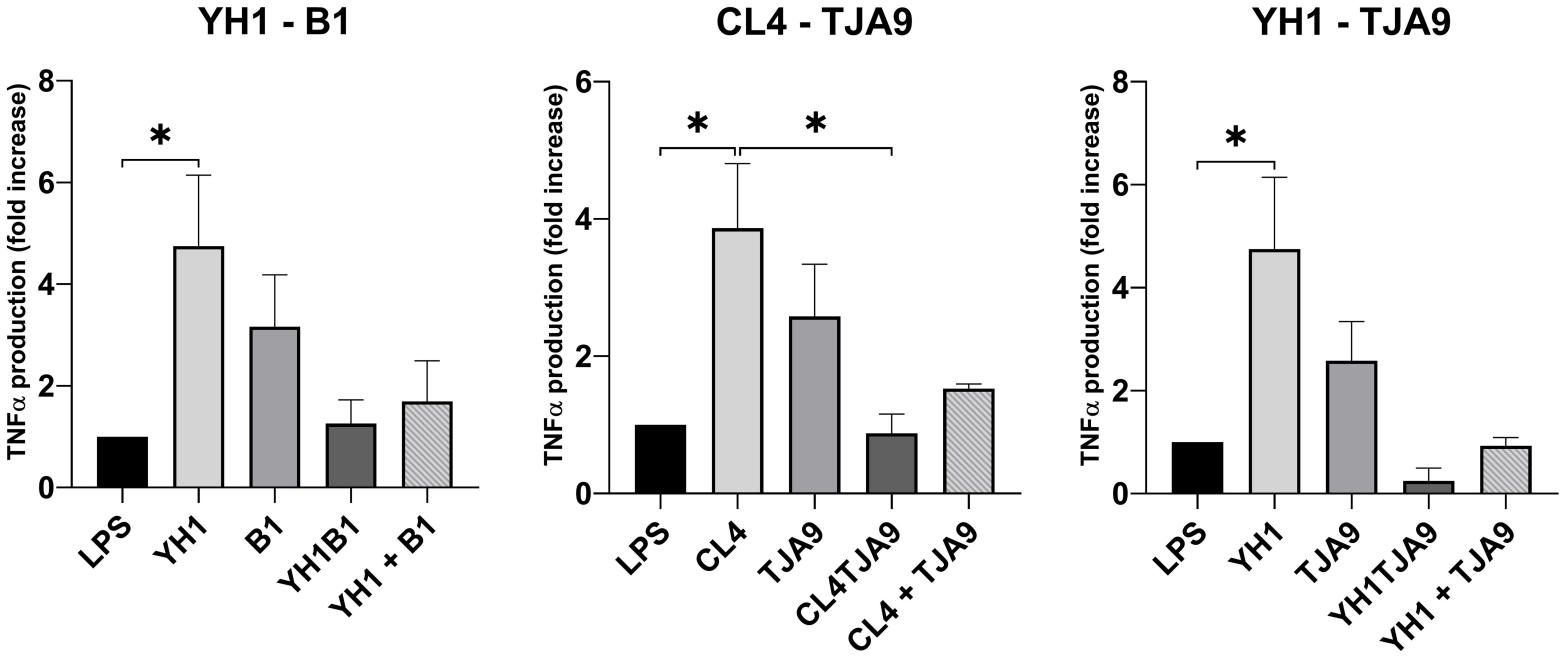
TNF-ɑ production by healthy human monocytes after stimulation with diverse single, co-cultures and mixtures of the selected yeast and lactobacillus strains and subsequent stimulation with LPS after 5 days, compared to stimulation with LPS only (LPS columns). Graphs show means and standard errors for 15 independent experiments (N = 15). Statistical significance was assessed by one-way ANOVA; * for p < 0.05.

**Figure 6:**
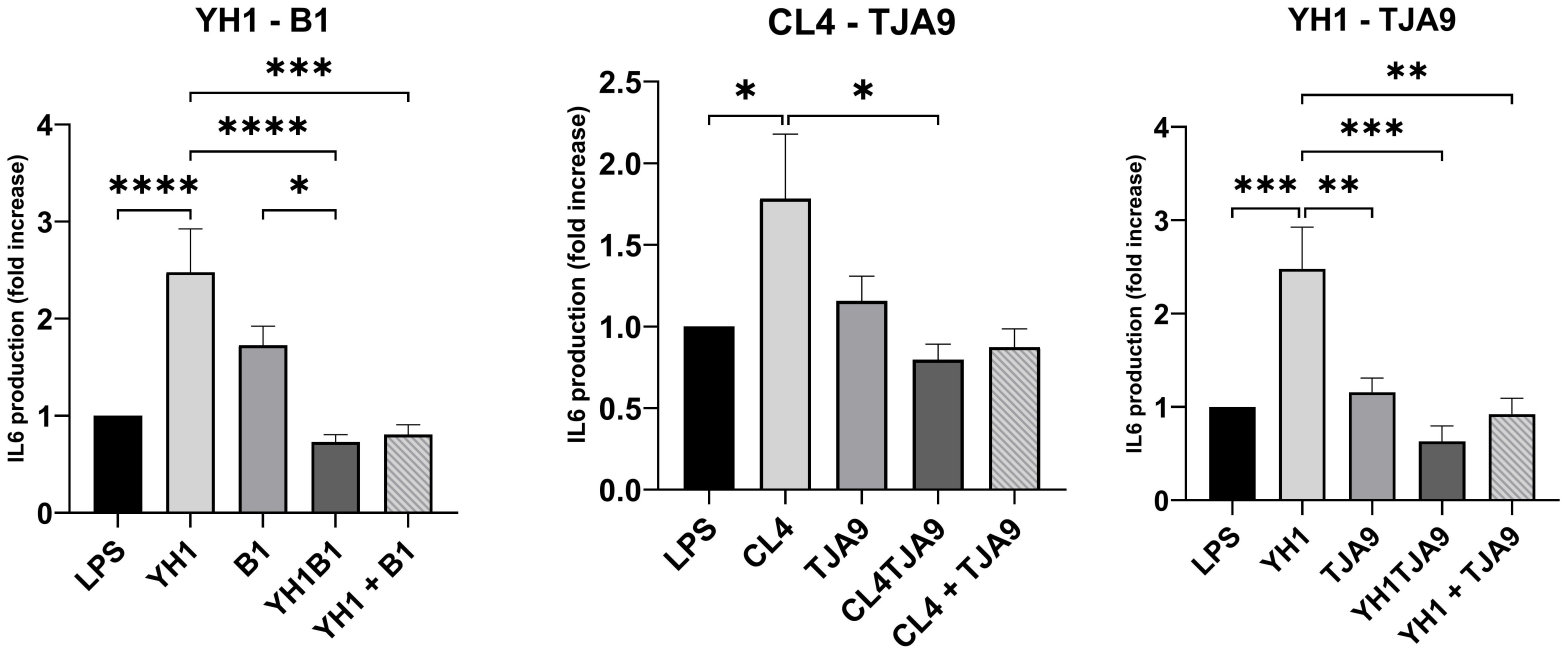
IL-6 production by healthy human monocytes after stimulation with diverse single, co-cultures and mixtures of the selected yeast and lactobacillus strains and subsequent stimulation with LPS after 5 days, compared to stimulation with LPS only (LPS columns). Cytokine production is expressed as fold increase compared to control. Graphs show means and standard errors for 15 independent experiments (N = 15). Statistical significance was assessed by one-way ANOVA; * for p < 0.05, ** for p < 0.01, *** for p < 0.001, **** for p < 0.0001.

Firstly, we assessed the ability of yeasts and lactobacilli, both in single and co-cultures, to stimulate the production of the pro-inflammatory cytokines TNF-ɑ (**Figure 5**) and IL-6 (**Figure 6**).

As depicted in **Figure 5**, both yeast strains have the ability to enhance TNF-ɑ production in monocytes after a second stimulus with LPS, compared with the control (LPS stimulus alone without the previous interaction with the microbe).

Neither the co-cultures nor the mixtures increased the TNF-ɑ production compared to the control. Interestingly, the co-cultures resulted in induced the fewest production of TNF-ɑ, showing a result comparable to the control in the case of CL4-TJA9 (both fermented milk).

**Figure 7:**
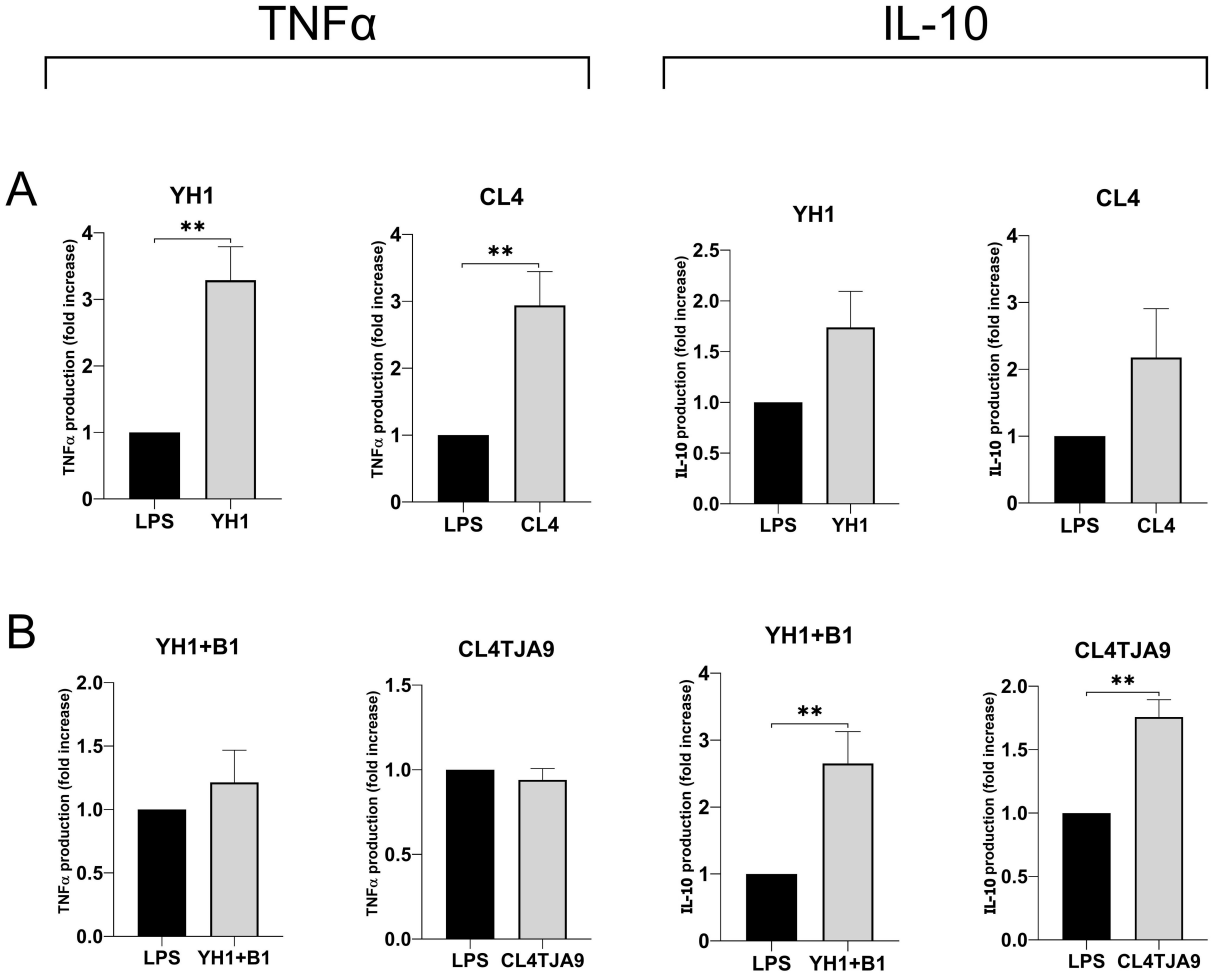
TNF-ɑ and IL-10 productions by human monocytes at Day 6 after 24-hour incubation with single culture **(A)**, co-cultures, and mixtures **(B)** of selected yeast and bacterial strains and a subsequent stimulus with LPS at Day 5. Graphs show means and standard errors for 6 independent experiments (N = 6). Statistical significance was assessed by the Mann-Whitney nonparametric test; ** for p < 0.01.

Similarly, the IL-6 production by yeasts in single cultures showed an increase compared to the control (**Figure 7**). In the same way as with TNF-ɑ, co-cultures did not stimulate a higher production of pro-inflammatory cytokines compared to the control. Here, each of the co-cultures resulted in statistically significant lower production of IL-6 compared to that produced by the single yeast culture, with results comparable to the control.

#### 3.3.2 Co-cultures and mixtures enhance anti-inflammatory responses

Since co-cultures did not induce the production of the pro-inflammatory cytokines IL-6 and TNF-ɑ, and in some cases a decreased production was observed compared to the control, we wanted to assess whether co-cultures and mixtures were able to induce the production of anti-inflammatory cytokines such as IL-10.

The most noticeable results, which refer to the production of the anti-inflammatory cytokine IL-10 and the pro-inflammatory TNF-ɑ, are shown in **Figure 7**, while the complete panels are available in **Supplementary Material** (**Supplementary Figure S6**).

Here we confirmed that single cultures yeast strains (**Figure 7A**) induce the production of the pro-inflammatory cytokine TNF-ɑ, whereas both the mixture of YH1 (Crohn’s disease) with B1 (commercial probiotic) and the co-culture of CL4 with TJA9 (both isolated from the fermented beverage) strongly increase the production of the anti-inflammatory cytokine IL-10 compared to the control (**Figure 7B**).

#### 3.3.3 Monocytes treated with co-cultures and mixtures show an anti-inflammatory immunophenotype

To understand if the cytokine profiles were linked to a change in the monocytes’ expressions of surface activation markers, an immunophenotype assay was performed in the same treatment conditions.

**Figure 8:**
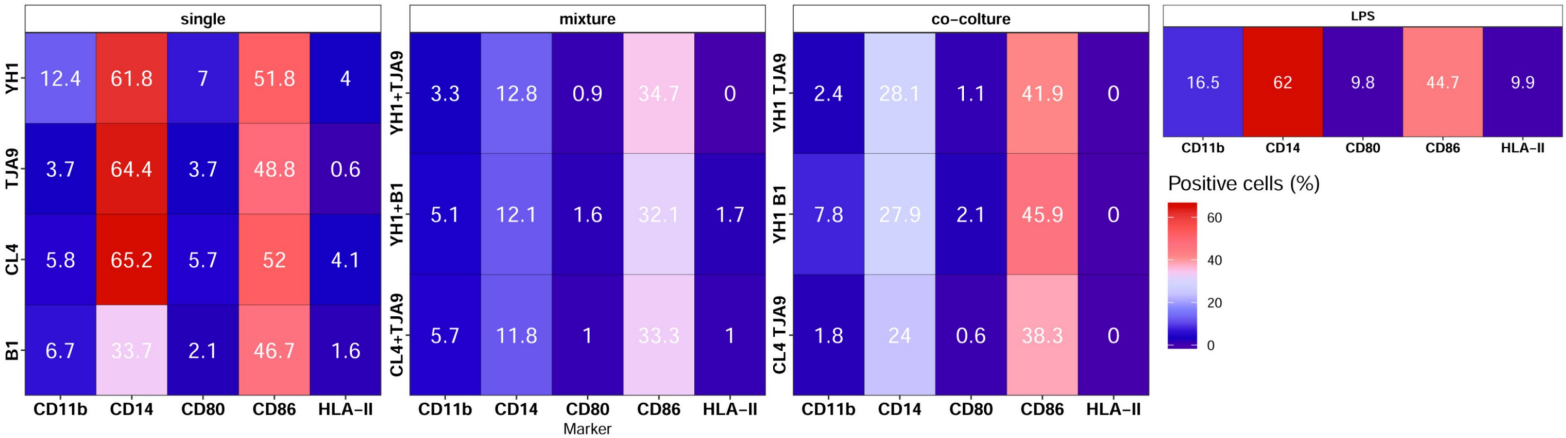
Surface markers pattern of human monocytes at Day 6 after 24-hour incubation with single culture, co-cultures, and mixtures of selected yeast and bacterial strains and a subsequent stimulus with LPS at Day 5. Results are expressed as the percentage of cells that showed a specific marker on their surface. Percentages of positive cells are reported on a color scale from the lower values (blue) to the higher ones (red).

Results of immunophenotyping (**Figure 8**) showed that monocytes treated with single cultures of both yeasts and lactobacilli present a surface marker profile resembling those of the control group, i.e. monocytes treated with LPS only. On the contrary, monocytes treated with both co-cultures and mixtures present decreased expression of markers CD14 and CD86.

## 4 Discussion

Our study explored the relationships within co-cultures of S. cerevisiae (strains YH1 and CL4) and lactobacilli (*Lactiplantibacillus plantarum*, strain B1, and *Lactobacillus delbrueckii*, strain TJA9). Firstly, we identified an increase in *L. plantarum* B1 growth yield when co-cultured with *S. cerevisiae*, but only with a yeast-bacterial cells ratio of 1:10 (which is the ratio commonly found in diverse ecological niches between lactobacilli and yeasts), whereas no changes in bacterial growth yield were observed in the 1:1 ratio. Several studies show the interactions between microbes depend on cellular density, whose increase is due to the increasing production of molecules related to the quorum sensing mechanism (67, 68). These results led us to hypothesize that the growth yield increase of *L. plantarum* only in the 1:10 ratio co-culture depends on quorum sensing-associated dynamics. During the observation of microbial growths on the optical microscope, we observed that the 1:10 ratio (and not 1:1) produced yeasts with swollen vacuoles, a marker of cell distress due to hypo-osmotic conditions or glucose deprivation (69). Presumably, yeast cells suffer when largely outnumbered by a bacterial presence. An alternative possible explanation for the growth yield increase of B1 relies on competition mechanisms exerted by some bacterial species (including *L. plantarum*) which have shown the ability to induce the prion [GAR+] in laboratory yeasts, reducing the yeast’s ability to ferment glucose (70).

On the contrary, the *L. debrueckii* TJA9 strain’s growth yield was reduced in the co-cultures with both yeast strains, suggesting that lactobacilli growth yield in co-cultures depends on the bacterial strain and that the increase of yeast growth yield was not related to the biological matrix of isolation.

We then investigated if yeast metabolites, produced in the co-culture with B1, could favor the growth of TJA9, by cultivating this species in the exhausted media of yeast-B1 co-cultures (data not shown). *L. delbrueckii* did not seem to benefit from the metabolites of the exhausted media, indicating the released metabolites did not modify its growth yield.

One of the goals of this study was to explore the potential of probiotic approaches for Inflammatory Bowel Diseases (IBD), conditions where patients typically show decreased levels of both SCFAs and SCFAs-producing microorganisms (71, 72). To achieve a broader understanding of the possible beneficial effects of yeast-lactobacilli communities, we included an evaluation of the SCFAs production in our investigation. We cultivated all the species, both as single and co-cultures, in a chemically defined medium previously used for the detection of metabolic products of yeast-bacterial co-cultures (14) with the scope of assessing the differences in SCFA’s production between strains and conditions through GC-MS. Taking into consideration the B1-CL4 and B1-YH1 co-cultures, results showed that the bacterial single culture produced fewer SCFAs than the other two conditions (yeast single culture and co-cultures), and that, with the exception of Valeric acid, all the SCFAs production increased over time. Another aspect to be noticed is a relatively higher production of 2-MethylButyric acid by B1-CL4 co-cultures with respect to both the single cultures. Butyric acid also showed the same results for the B1-YH1 co-culture. Despite providing insights into the metabolic production dynamics between yeast and lactobacilli communities, the overall SCFAs production resulted in small amounts. This outcome can be convincingly explained by the fact that yeast and lactobacilli are not great producers of SCFAs when compared to other members of the human microbiota, such as bacteria of the Bifidobacteriaceae, Lachnospiraceae, Prevotellaceae and Ruminococcaceae families (73, 74).

Together with SCFAs-producing microorganisms, also commensal fungi have been shown to play a crucial role in IBD pathogenesis and chronicity (35, 36, 75–78). It is worth considering that the first clues on the potential involvement of fungi in IBD came from the observation of an abnormal response to *S. cerevisiae* in Crohn’s disease (CD) patients (47). In recent years, Sokol and colleagues observed a reduction of *S. cerevisiae* proportion in CD patients compared with healthy subjects (75). In contrast, Liguori and colleagues observed that *S. cerevisiae* was enriched in CD patients’ non-inflamed gut mucosa (77). On the other hand, a recent study reported that *S. cerevisiae* can exacerbate colitis and affect gut barrier permeability (78). All these studies have highlighted the relevance of *S. cerevisiae* in gut inflammation but with controversial outcomes. The wide genetic and phenotypic variability observed for *S. cerevisiae* (48, 60) could explain the inconsistencies in the results of different studies. Therefore, it is likely that both the strain-specificity and the multispecies cross-kingdom interactions taking place in the gut are associated with different patterns of immunomodulation, balancing inflammatory and tolerogenic responses.

Yeasts have been shown to interact with bacteria and other yeasts also through the activation of innate immune responses such as “trained immunity” (51,52, 57). To gain further insight into the immune consequences of our findings, the immunomodulatory potential of lactobacilli and yeasts in single and co-cultures was assessed through multiple immunological assays on human monocytes *in vitro*. Single cultures of both lactobacilli and yeasts induced a significantly higher production of the pro-inflammatory cytokines TNF-ɑ and IL-6, showing great potential as inducers of trained immunity in human monocytes.

The mixture of the lactobacillus B1 and the yeast YH1, as well as the co-culture of the lactobacillus TJA9 and the yeast CL4 (both isolated from the fermented milk), induced markedly increased production of the anti-inflammatory cytokine IL-10, suggesting a role in dampening the inflammation response. Given the complex composition of the gut microbiota, it is conceivable that the joint activation of receptors for both yeast and bacterial commensal species induces an immune response towards tolerance, resembling a common situation in the gut. In contrast, the exclusive activation of specific receptors for either bacteria or yeasts induces an inflammatory response. These results were consistent with the monocytes’ surface markers activation profiles assessed through immunophenotype assays. In fact, monocytes treated with single cultures of bacteria or yeasts presented a profile similar to that of the control group (monocytes treated with LPS only), showing a high activation. When monocytes were treated with mixtures or co-cultures, they showed a reduction in the expression of CD14 and CD86. CD14 is implicated in the recognition of LPS, while CD86 is crucial for the activation of T cells (49, 79, 80). Reduction in the expression of both these receptors could lead to the induction of a tolerance response towards the co-presence of bacteria and yeasts as harmless organisms.

This study took an explorative approach to investigate community dynamics between lactobacilli and yeasts. The results demonstrate that the nature of the interaction, the strains involved, and the concentrations of the cells are crucial factors in determining the outcome in terms of growth yield, metabolic products, and immunomodulatory effects. While single cultures of yeasts and lactobacilli appear to be ideal candidates for developing immune-enhancing products, probiotics containing co-cultures of yeasts and lactobacilli appear as useful tools to induce tolerogenic responses on the same immune cells both in terms of cytokine profiles and activation surface markers.

Since this study was based on *in vitro* interactions between two strains at a time, there are some areas for improvement. Future studies could employ three-dimensional models of reconstituted intestinal tissue and richer microbial communities to obtain results that are more representative of strain interactions in an *in vivo* system. At the same time, our findings, thanks to the use of yeast and lactobacilli strains that can be part of the human gut microbiota and the broad-spectrum analysis of their interactions, open new possibilities in the design of probiotic and dietary therapies.

## Data Availability

The raw data supporting the conclusions of this article will be made available by the authors, without undue reservation.
