# Supplementary material for: Impact of cooperative or competitive dynamics between the yeast Saccharomyces cerevisiae and lactobacilli on the immune response of the host
Source: Front Immunol. 2024 Oct 10;15:1399842. doi: 10.3389/fimmu.2024.1399842 (PMC11499123; doi:10.3389/fimmu.2024.1399842)
Supplement: Supplementary file 1 [file Presentation1.pptx]

## Slide 1
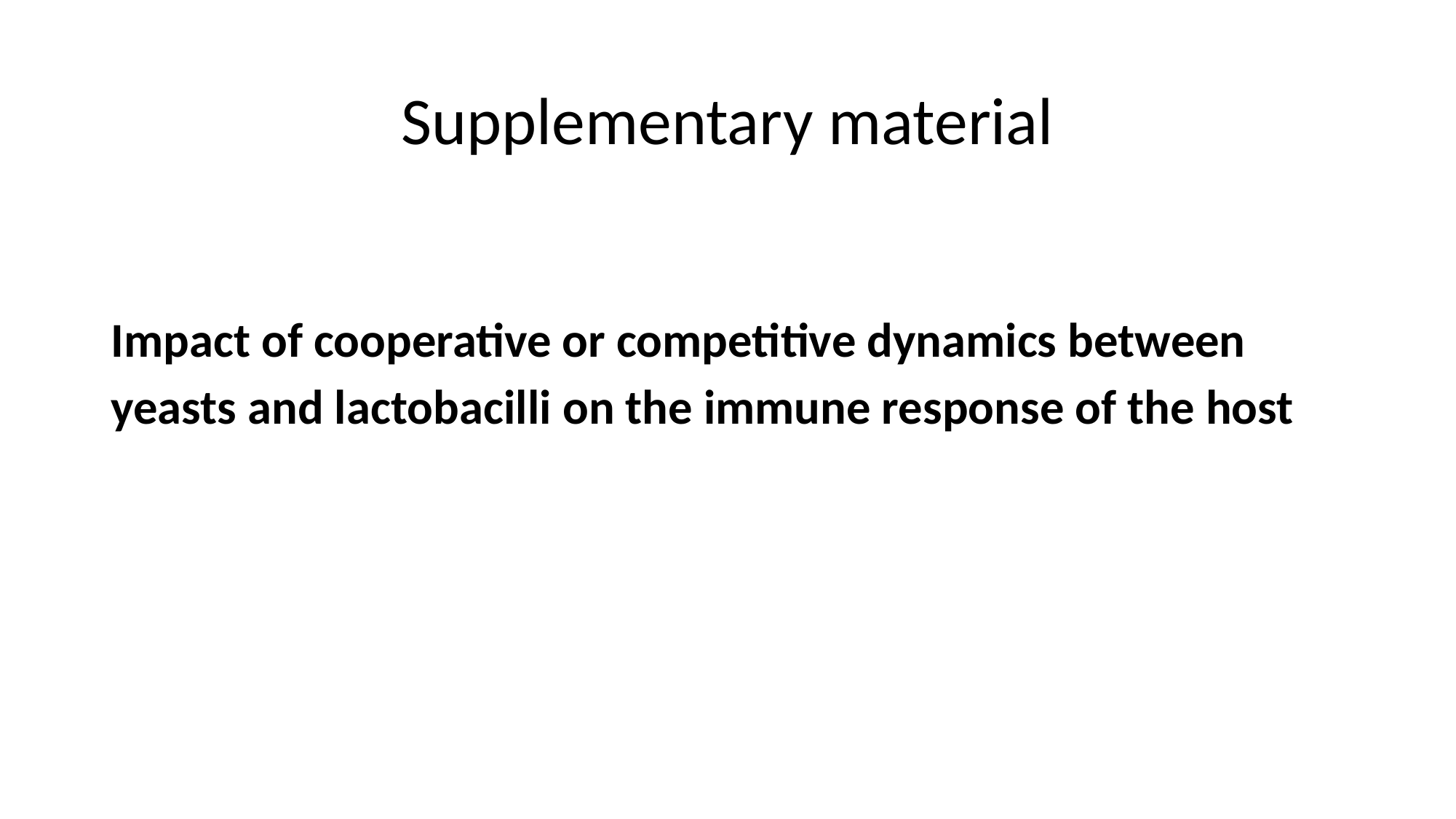

# Supplementary material
Impact of cooperative or competitive dynamics between yeasts and lactobacilli on the immune response of the host

## Slide 2
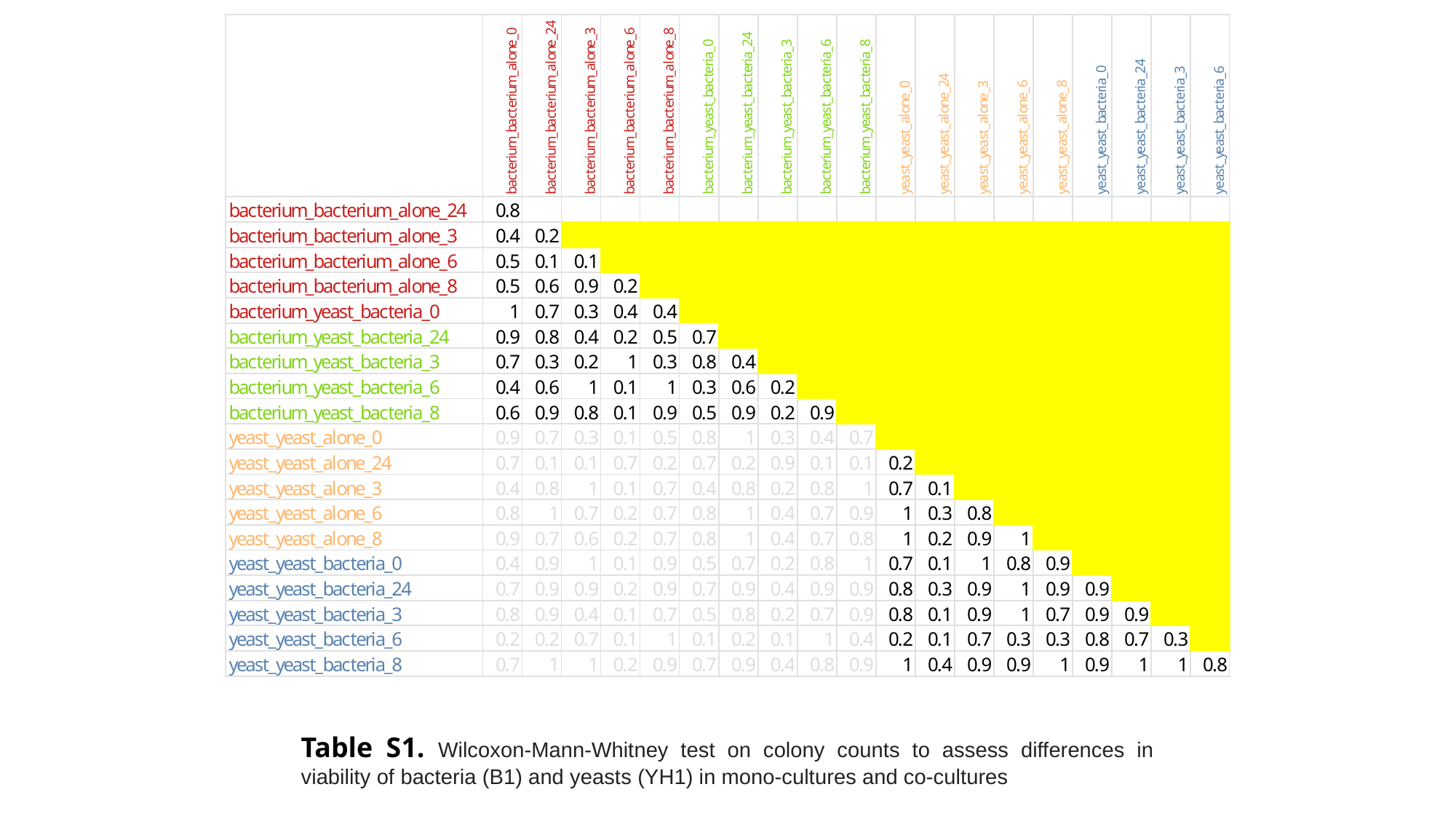

Table S1. Wilcoxon-Mann-Whitney test on colony counts to assess differences in viability of bacteria (B1) and yeasts (YH1) in mono-cultures and co-cultures

## Slide 3
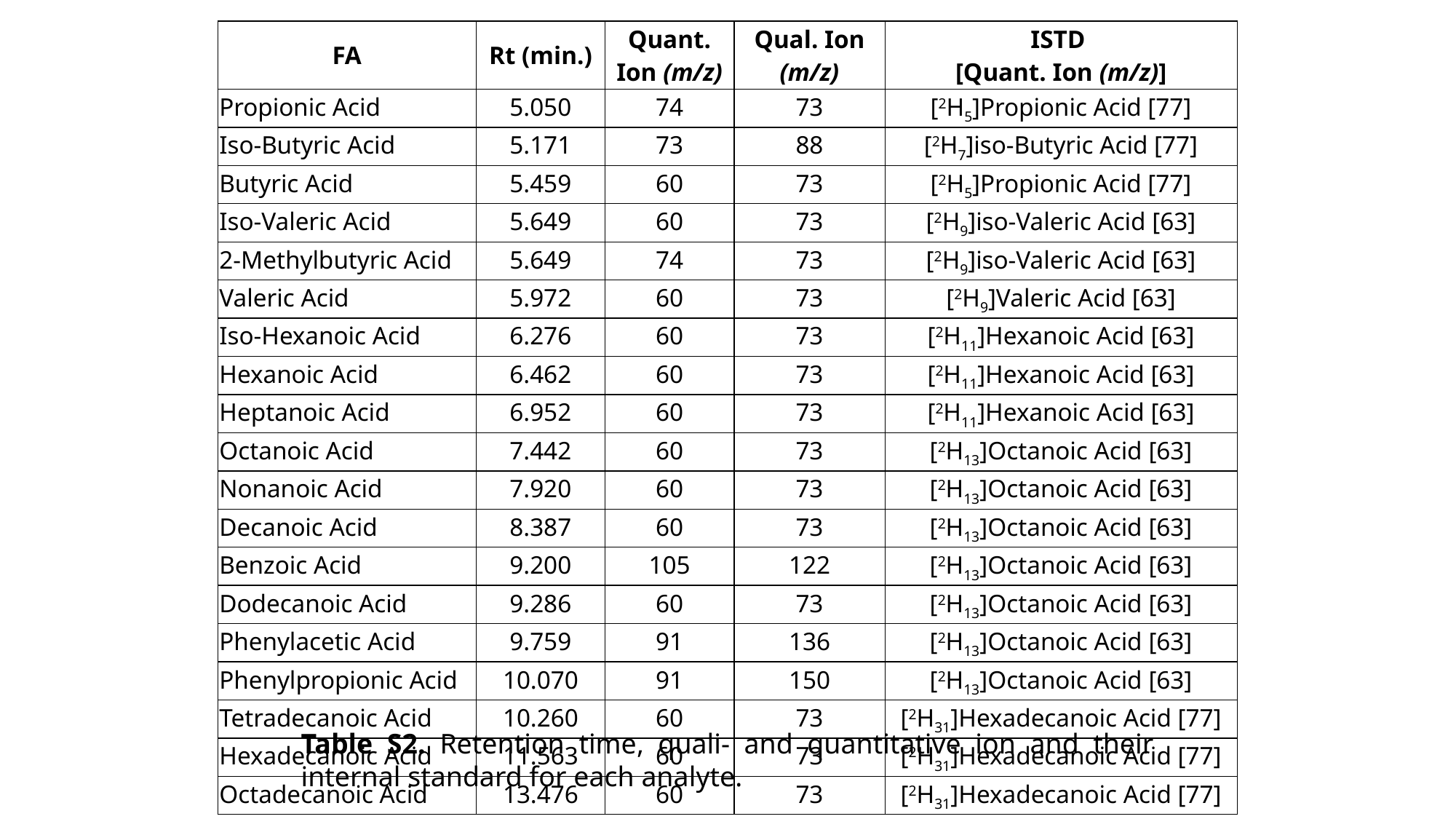

| FA | Rt (min.) | Quant. Ion (m/z) | Qual. Ion (m/z) | ISTD  [Quant. Ion (m/z)] |
| --- | --- | --- | --- | --- |
| Propionic Acid | 5.050 | 74 | 73 | [2H5]Propionic Acid [77] |
| Iso-Butyric Acid | 5.171 | 73 | 88 | [2H7]iso-Butyric Acid [77] |
| Butyric Acid | 5.459 | 60 | 73 | [2H5]Propionic Acid [77] |
| Iso-Valeric Acid | 5.649 | 60 | 73 | [2H9]iso-Valeric Acid [63] |
| 2-Methylbutyric Acid | 5.649 | 74 | 73 | [2H9]iso-Valeric Acid [63] |
| Valeric Acid | 5.972 | 60 | 73 | [2H9]Valeric Acid [63] |
| Iso-Hexanoic Acid | 6.276 | 60 | 73 | [2H11]Hexanoic Acid [63] |
| Hexanoic Acid | 6.462 | 60 | 73 | [2H11]Hexanoic Acid [63] |
| Heptanoic Acid | 6.952 | 60 | 73 | [2H11]Hexanoic Acid [63] |
| Octanoic Acid | 7.442 | 60 | 73 | [2H13]Octanoic Acid [63] |
| Nonanoic Acid | 7.920 | 60 | 73 | [2H13]Octanoic Acid [63] |
| Decanoic Acid | 8.387 | 60 | 73 | [2H13]Octanoic Acid [63] |
| Benzoic Acid | 9.200 | 105 | 122 | [2H13]Octanoic Acid [63] |
| Dodecanoic Acid | 9.286 | 60 | 73 | [2H13]Octanoic Acid [63] |
| Phenylacetic Acid | 9.759 | 91 | 136 | [2H13]Octanoic Acid [63] |
| Phenylpropionic Acid | 10.070 | 91 | 150 | [2H13]Octanoic Acid [63] |
| Tetradecanoic Acid | 10.260 | 60 | 73 | [2H31]Hexadecanoic Acid [77] |
| Hexadecanoic Acid | 11.563 | 60 | 73 | [2H31]Hexadecanoic Acid [77] |
| Octadecanoic Acid | 13.476 | 60 | 73 | [2H31]Hexadecanoic Acid [77] |
Table S2. Retention time, quali- and quantitative ion and their internal standard for each analyte.

## Slide 4
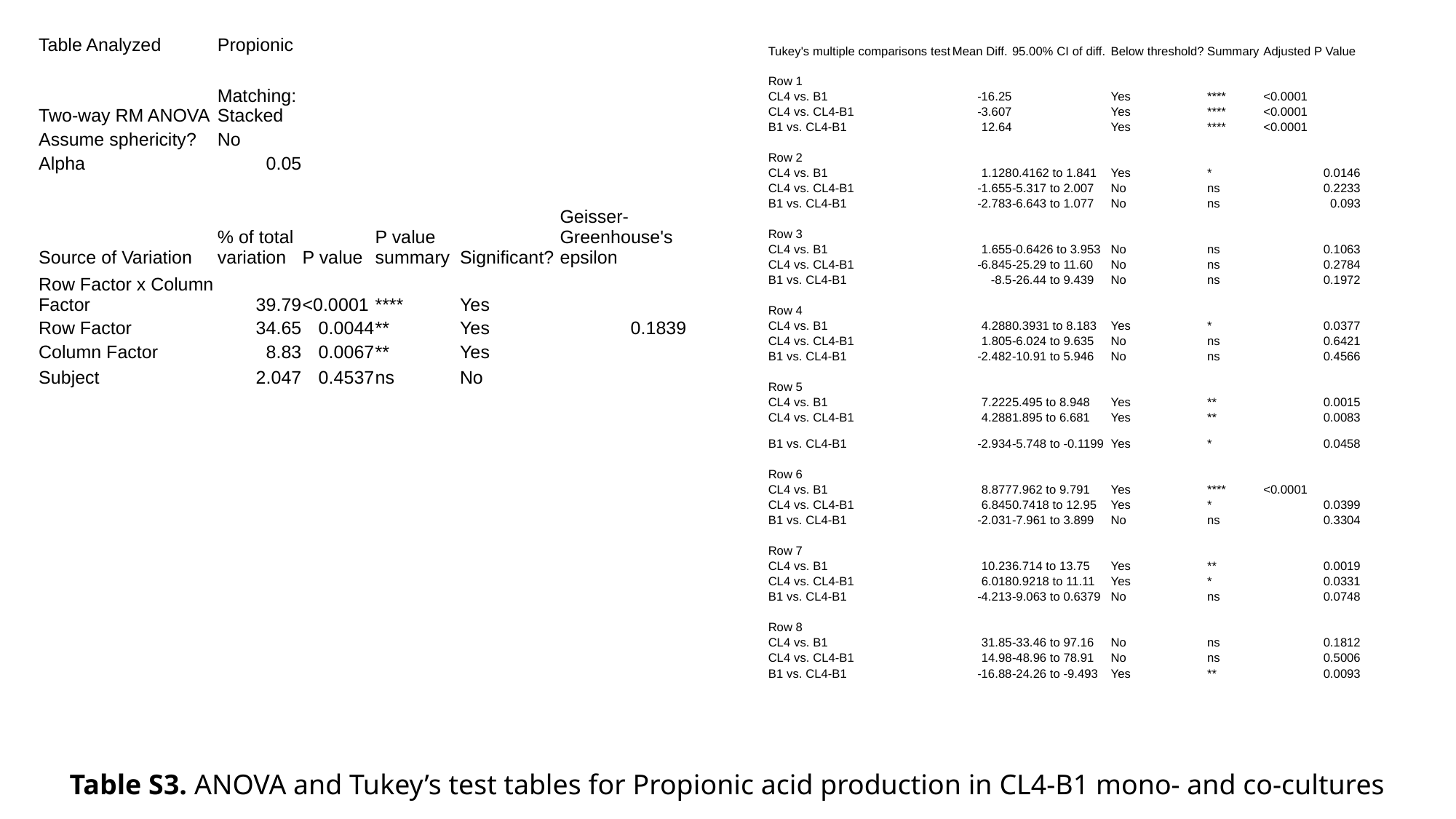

| Table Analyzed | Propionic | | | | |
| --- | --- | --- | --- | --- | --- |
| | | | | | |
| Two-way RM ANOVA | Matching: Stacked | | | | |
| Assume sphericity? | No | | | | |
| Alpha | 0.05 | | | | |
| | | | | | |
| Source of Variation | % of total variation | P value | P value summary | Significant? | Geisser-Greenhouse's epsilon |
| Row Factor x Column Factor | 39.79 | <0.0001 | \*\*\*\* | Yes | |
| Row Factor | 34.65 | 0.0044 | \*\* | Yes | 0.1839 |
| Column Factor | 8.83 | 0.0067 | \*\* | Yes | |
| Subject | 2.047 | 0.4537 | ns | No | |
| Tukey's multiple comparisons test | Mean Diff. | 95.00% CI of diff. | Below threshold? | Summary | Adjusted P Value |
| --- | --- | --- | --- | --- | --- |
| | | | | | |
| Row 1 | | | | | |
| CL4 vs. B1 | -16.25 | | Yes | \*\*\*\* | <0.0001 |
| CL4 vs. CL4-B1 | -3.607 | | Yes | \*\*\*\* | <0.0001 |
| B1 vs. CL4-B1 | 12.64 | | Yes | \*\*\*\* | <0.0001 |
| | | | | | |
| Row 2 | | | | | |
| CL4 vs. B1 | 1.128 | 0.4162 to 1.841 | Yes | \* | 0.0146 |
| CL4 vs. CL4-B1 | -1.655 | -5.317 to 2.007 | No | ns | 0.2233 |
| B1 vs. CL4-B1 | -2.783 | -6.643 to 1.077 | No | ns | 0.093 |
| | | | | | |
| Row 3 | | | | | |
| CL4 vs. B1 | 1.655 | -0.6426 to 3.953 | No | ns | 0.1063 |
| CL4 vs. CL4-B1 | -6.845 | -25.29 to 11.60 | No | ns | 0.2784 |
| B1 vs. CL4-B1 | -8.5 | -26.44 to 9.439 | No | ns | 0.1972 |
| | | | | | |
| Row 4 | | | | | |
| CL4 vs. B1 | 4.288 | 0.3931 to 8.183 | Yes | \* | 0.0377 |
| CL4 vs. CL4-B1 | 1.805 | -6.024 to 9.635 | No | ns | 0.6421 |
| B1 vs. CL4-B1 | -2.482 | -10.91 to 5.946 | No | ns | 0.4566 |
| | | | | | |
| Row 5 | | | | | |
| CL4 vs. B1 | 7.222 | 5.495 to 8.948 | Yes | \*\* | 0.0015 |
| CL4 vs. CL4-B1 | 4.288 | 1.895 to 6.681 | Yes | \*\* | 0.0083 |
| B1 vs. CL4-B1 | -2.934 | -5.748 to -0.1199 | Yes | \* | 0.0458 |
| | | | | | |
| Row 6 | | | | | |
| CL4 vs. B1 | 8.877 | 7.962 to 9.791 | Yes | \*\*\*\* | <0.0001 |
| CL4 vs. CL4-B1 | 6.845 | 0.7418 to 12.95 | Yes | \* | 0.0399 |
| B1 vs. CL4-B1 | -2.031 | -7.961 to 3.899 | No | ns | 0.3304 |
| | | | | | |
| Row 7 | | | | | |
| CL4 vs. B1 | 10.23 | 6.714 to 13.75 | Yes | \*\* | 0.0019 |
| CL4 vs. CL4-B1 | 6.018 | 0.9218 to 11.11 | Yes | \* | 0.0331 |
| B1 vs. CL4-B1 | -4.213 | -9.063 to 0.6379 | No | ns | 0.0748 |
| | | | | | |
| Row 8 | | | | | |
| CL4 vs. B1 | 31.85 | -33.46 to 97.16 | No | ns | 0.1812 |
| CL4 vs. CL4-B1 | 14.98 | -48.96 to 78.91 | No | ns | 0.5006 |
| B1 vs. CL4-B1 | -16.88 | -24.26 to -9.493 | Yes | \*\* | 0.0093 |
Table S3. ANOVA and Tukey’s test tables for Propionic acid production in CL4-B1 mono- and co-cultures

## Slide 5
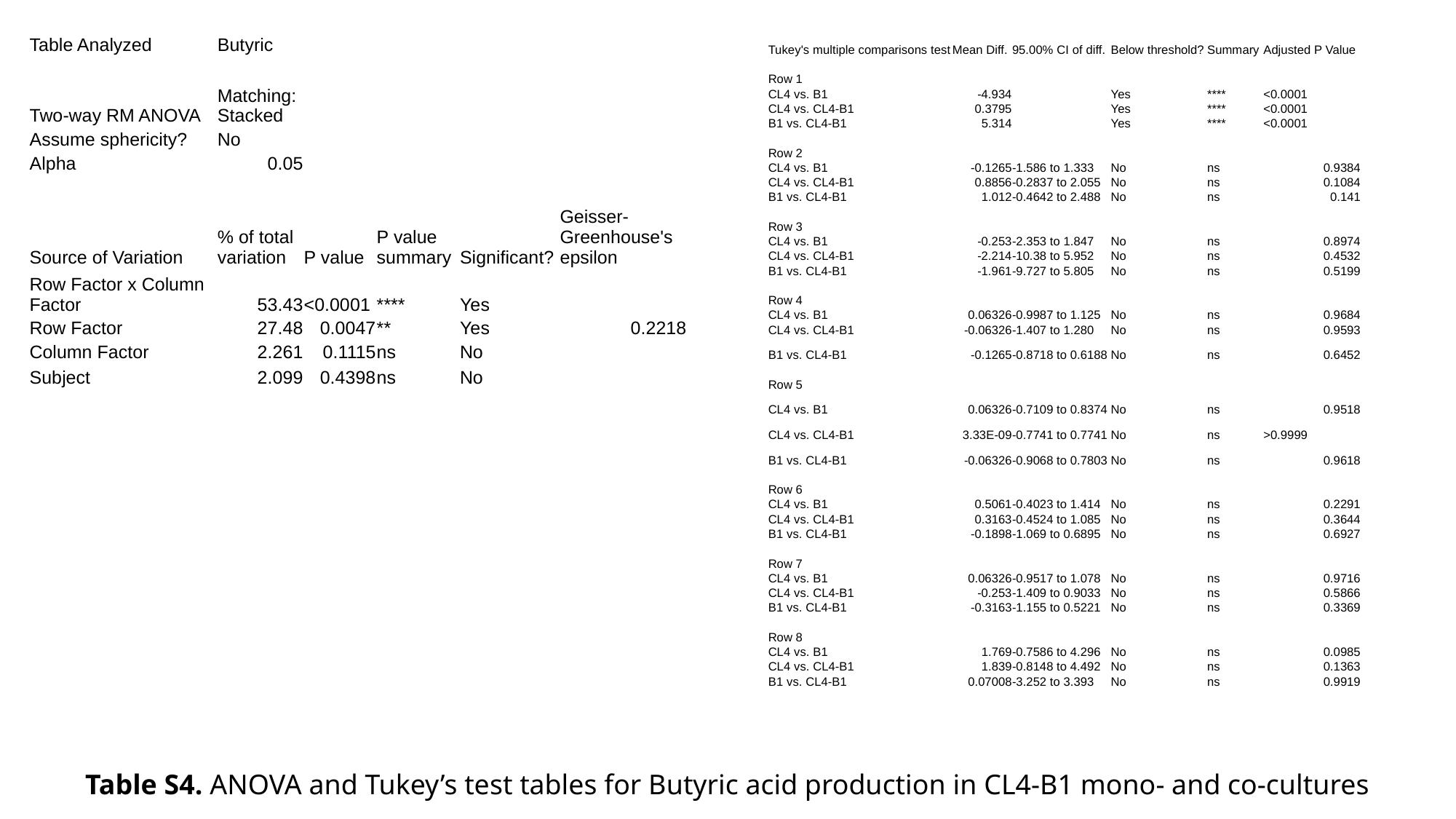

| Table Analyzed | Butyric | | | | |
| --- | --- | --- | --- | --- | --- |
| | | | | | |
| Two-way RM ANOVA | Matching: Stacked | | | | |
| Assume sphericity? | No | | | | |
| Alpha | 0.05 | | | | |
| | | | | | |
| Source of Variation | % of total variation | P value | P value summary | Significant? | Geisser-Greenhouse's epsilon |
| Row Factor x Column Factor | 53.43 | <0.0001 | \*\*\*\* | Yes | |
| Row Factor | 27.48 | 0.0047 | \*\* | Yes | 0.2218 |
| Column Factor | 2.261 | 0.1115 | ns | No | |
| Subject | 2.099 | 0.4398 | ns | No | |
| Tukey's multiple comparisons test | Mean Diff. | 95.00% CI of diff. | Below threshold? | Summary | Adjusted P Value |
| --- | --- | --- | --- | --- | --- |
| | | | | | |
| Row 1 | | | | | |
| CL4 vs. B1 | -4.934 | | Yes | \*\*\*\* | <0.0001 |
| CL4 vs. CL4-B1 | 0.3795 | | Yes | \*\*\*\* | <0.0001 |
| B1 vs. CL4-B1 | 5.314 | | Yes | \*\*\*\* | <0.0001 |
| | | | | | |
| Row 2 | | | | | |
| CL4 vs. B1 | -0.1265 | -1.586 to 1.333 | No | ns | 0.9384 |
| CL4 vs. CL4-B1 | 0.8856 | -0.2837 to 2.055 | No | ns | 0.1084 |
| B1 vs. CL4-B1 | 1.012 | -0.4642 to 2.488 | No | ns | 0.141 |
| | | | | | |
| Row 3 | | | | | |
| CL4 vs. B1 | -0.253 | -2.353 to 1.847 | No | ns | 0.8974 |
| CL4 vs. CL4-B1 | -2.214 | -10.38 to 5.952 | No | ns | 0.4532 |
| B1 vs. CL4-B1 | -1.961 | -9.727 to 5.805 | No | ns | 0.5199 |
| | | | | | |
| Row 4 | | | | | |
| CL4 vs. B1 | 0.06326 | -0.9987 to 1.125 | No | ns | 0.9684 |
| CL4 vs. CL4-B1 | -0.06326 | -1.407 to 1.280 | No | ns | 0.9593 |
| B1 vs. CL4-B1 | -0.1265 | -0.8718 to 0.6188 | No | ns | 0.6452 |
| | | | | | |
| Row 5 | | | | | |
| CL4 vs. B1 | 0.06326 | -0.7109 to 0.8374 | No | ns | 0.9518 |
| CL4 vs. CL4-B1 | 3.33E-09 | -0.7741 to 0.7741 | No | ns | >0.9999 |
| B1 vs. CL4-B1 | -0.06326 | -0.9068 to 0.7803 | No | ns | 0.9618 |
| | | | | | |
| Row 6 | | | | | |
| CL4 vs. B1 | 0.5061 | -0.4023 to 1.414 | No | ns | 0.2291 |
| CL4 vs. CL4-B1 | 0.3163 | -0.4524 to 1.085 | No | ns | 0.3644 |
| B1 vs. CL4-B1 | -0.1898 | -1.069 to 0.6895 | No | ns | 0.6927 |
| | | | | | |
| Row 7 | | | | | |
| CL4 vs. B1 | 0.06326 | -0.9517 to 1.078 | No | ns | 0.9716 |
| CL4 vs. CL4-B1 | -0.253 | -1.409 to 0.9033 | No | ns | 0.5866 |
| B1 vs. CL4-B1 | -0.3163 | -1.155 to 0.5221 | No | ns | 0.3369 |
| | | | | | |
| Row 8 | | | | | |
| CL4 vs. B1 | 1.769 | -0.7586 to 4.296 | No | ns | 0.0985 |
| CL4 vs. CL4-B1 | 1.839 | -0.8148 to 4.492 | No | ns | 0.1363 |
| B1 vs. CL4-B1 | 0.07008 | -3.252 to 3.393 | No | ns | 0.9919 |
Table S4. ANOVA and Tukey’s test tables for Butyric acid production in CL4-B1 mono- and co-cultures

## Slide 6
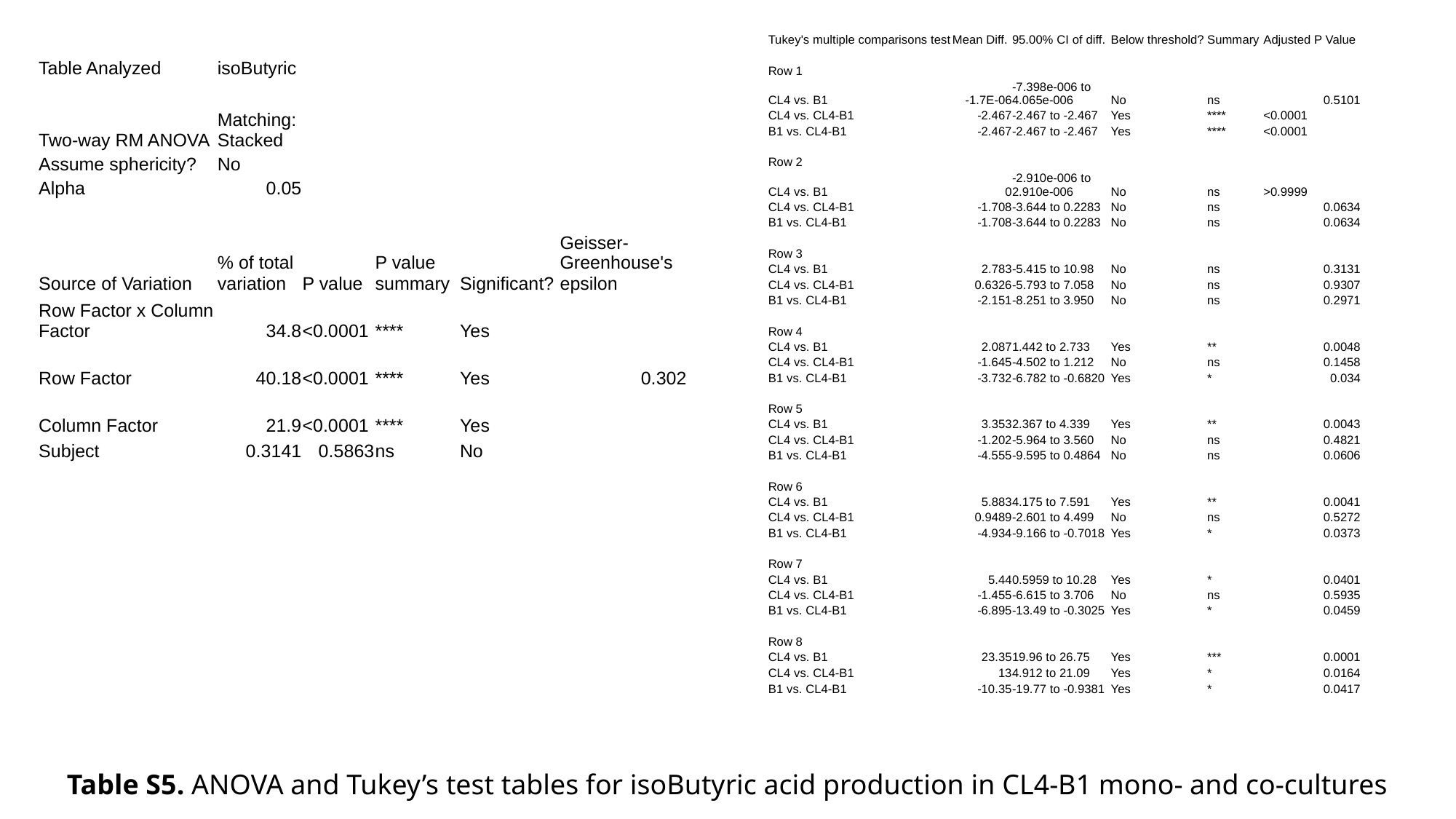

| Table Analyzed | isoButyric | | | | |
| --- | --- | --- | --- | --- | --- |
| | | | | | |
| Two-way RM ANOVA | Matching: Stacked | | | | |
| Assume sphericity? | No | | | | |
| Alpha | 0.05 | | | | |
| | | | | | |
| Source of Variation | % of total variation | P value | P value summary | Significant? | Geisser-Greenhouse's epsilon |
| Row Factor x Column Factor | 34.8 | <0.0001 | \*\*\*\* | Yes | |
| Row Factor | 40.18 | <0.0001 | \*\*\*\* | Yes | 0.302 |
| Column Factor | 21.9 | <0.0001 | \*\*\*\* | Yes | |
| Subject | 0.3141 | 0.5863 | ns | No | |
| Tukey's multiple comparisons test | Mean Diff. | 95.00% CI of diff. | Below threshold? | Summary | Adjusted P Value |
| --- | --- | --- | --- | --- | --- |
| | | | | | |
| Row 1 | | | | | |
| CL4 vs. B1 | -1.7E-06 | -7.398e-006 to 4.065e-006 | No | ns | 0.5101 |
| CL4 vs. CL4-B1 | -2.467 | -2.467 to -2.467 | Yes | \*\*\*\* | <0.0001 |
| B1 vs. CL4-B1 | -2.467 | -2.467 to -2.467 | Yes | \*\*\*\* | <0.0001 |
| | | | | | |
| Row 2 | | | | | |
| CL4 vs. B1 | 0 | -2.910e-006 to 2.910e-006 | No | ns | >0.9999 |
| CL4 vs. CL4-B1 | -1.708 | -3.644 to 0.2283 | No | ns | 0.0634 |
| B1 vs. CL4-B1 | -1.708 | -3.644 to 0.2283 | No | ns | 0.0634 |
| | | | | | |
| Row 3 | | | | | |
| CL4 vs. B1 | 2.783 | -5.415 to 10.98 | No | ns | 0.3131 |
| CL4 vs. CL4-B1 | 0.6326 | -5.793 to 7.058 | No | ns | 0.9307 |
| B1 vs. CL4-B1 | -2.151 | -8.251 to 3.950 | No | ns | 0.2971 |
| | | | | | |
| Row 4 | | | | | |
| CL4 vs. B1 | 2.087 | 1.442 to 2.733 | Yes | \*\* | 0.0048 |
| CL4 vs. CL4-B1 | -1.645 | -4.502 to 1.212 | No | ns | 0.1458 |
| B1 vs. CL4-B1 | -3.732 | -6.782 to -0.6820 | Yes | \* | 0.034 |
| | | | | | |
| Row 5 | | | | | |
| CL4 vs. B1 | 3.353 | 2.367 to 4.339 | Yes | \*\* | 0.0043 |
| CL4 vs. CL4-B1 | -1.202 | -5.964 to 3.560 | No | ns | 0.4821 |
| B1 vs. CL4-B1 | -4.555 | -9.595 to 0.4864 | No | ns | 0.0606 |
| | | | | | |
| Row 6 | | | | | |
| CL4 vs. B1 | 5.883 | 4.175 to 7.591 | Yes | \*\* | 0.0041 |
| CL4 vs. CL4-B1 | 0.9489 | -2.601 to 4.499 | No | ns | 0.5272 |
| B1 vs. CL4-B1 | -4.934 | -9.166 to -0.7018 | Yes | \* | 0.0373 |
| | | | | | |
| Row 7 | | | | | |
| CL4 vs. B1 | 5.44 | 0.5959 to 10.28 | Yes | \* | 0.0401 |
| CL4 vs. CL4-B1 | -1.455 | -6.615 to 3.706 | No | ns | 0.5935 |
| B1 vs. CL4-B1 | -6.895 | -13.49 to -0.3025 | Yes | \* | 0.0459 |
| | | | | | |
| Row 8 | | | | | |
| CL4 vs. B1 | 23.35 | 19.96 to 26.75 | Yes | \*\*\* | 0.0001 |
| CL4 vs. CL4-B1 | 13 | 4.912 to 21.09 | Yes | \* | 0.0164 |
| B1 vs. CL4-B1 | -10.35 | -19.77 to -0.9381 | Yes | \* | 0.0417 |
Table S5. ANOVA and Tukey’s test tables for isoButyric acid production in CL4-B1 mono- and co-cultures

## Slide 7
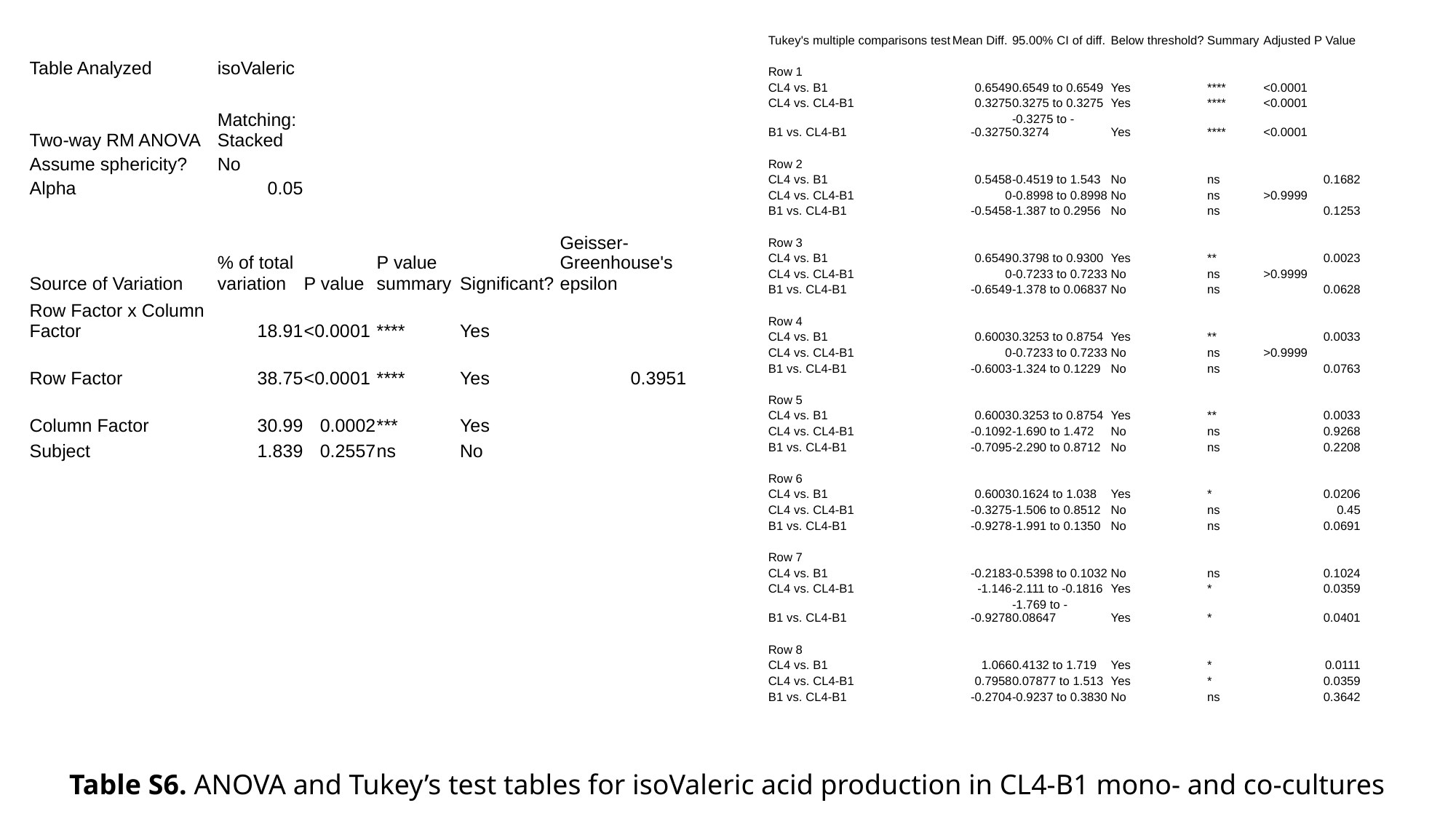

| Table Analyzed | isoValeric | | | | |
| --- | --- | --- | --- | --- | --- |
| | | | | | |
| Two-way RM ANOVA | Matching: Stacked | | | | |
| Assume sphericity? | No | | | | |
| Alpha | 0.05 | | | | |
| | | | | | |
| Source of Variation | % of total variation | P value | P value summary | Significant? | Geisser-Greenhouse's epsilon |
| Row Factor x Column Factor | 18.91 | <0.0001 | \*\*\*\* | Yes | |
| Row Factor | 38.75 | <0.0001 | \*\*\*\* | Yes | 0.3951 |
| Column Factor | 30.99 | 0.0002 | \*\*\* | Yes | |
| Subject | 1.839 | 0.2557 | ns | No | |
| Tukey's multiple comparisons test | Mean Diff. | 95.00% CI of diff. | Below threshold? | Summary | Adjusted P Value |
| --- | --- | --- | --- | --- | --- |
| | | | | | |
| Row 1 | | | | | |
| CL4 vs. B1 | 0.6549 | 0.6549 to 0.6549 | Yes | \*\*\*\* | <0.0001 |
| CL4 vs. CL4-B1 | 0.3275 | 0.3275 to 0.3275 | Yes | \*\*\*\* | <0.0001 |
| B1 vs. CL4-B1 | -0.3275 | -0.3275 to -0.3274 | Yes | \*\*\*\* | <0.0001 |
| | | | | | |
| Row 2 | | | | | |
| CL4 vs. B1 | 0.5458 | -0.4519 to 1.543 | No | ns | 0.1682 |
| CL4 vs. CL4-B1 | 0 | -0.8998 to 0.8998 | No | ns | >0.9999 |
| B1 vs. CL4-B1 | -0.5458 | -1.387 to 0.2956 | No | ns | 0.1253 |
| | | | | | |
| Row 3 | | | | | |
| CL4 vs. B1 | 0.6549 | 0.3798 to 0.9300 | Yes | \*\* | 0.0023 |
| CL4 vs. CL4-B1 | 0 | -0.7233 to 0.7233 | No | ns | >0.9999 |
| B1 vs. CL4-B1 | -0.6549 | -1.378 to 0.06837 | No | ns | 0.0628 |
| | | | | | |
| Row 4 | | | | | |
| CL4 vs. B1 | 0.6003 | 0.3253 to 0.8754 | Yes | \*\* | 0.0033 |
| CL4 vs. CL4-B1 | 0 | -0.7233 to 0.7233 | No | ns | >0.9999 |
| B1 vs. CL4-B1 | -0.6003 | -1.324 to 0.1229 | No | ns | 0.0763 |
| | | | | | |
| Row 5 | | | | | |
| CL4 vs. B1 | 0.6003 | 0.3253 to 0.8754 | Yes | \*\* | 0.0033 |
| CL4 vs. CL4-B1 | -0.1092 | -1.690 to 1.472 | No | ns | 0.9268 |
| B1 vs. CL4-B1 | -0.7095 | -2.290 to 0.8712 | No | ns | 0.2208 |
| | | | | | |
| Row 6 | | | | | |
| CL4 vs. B1 | 0.6003 | 0.1624 to 1.038 | Yes | \* | 0.0206 |
| CL4 vs. CL4-B1 | -0.3275 | -1.506 to 0.8512 | No | ns | 0.45 |
| B1 vs. CL4-B1 | -0.9278 | -1.991 to 0.1350 | No | ns | 0.0691 |
| | | | | | |
| Row 7 | | | | | |
| CL4 vs. B1 | -0.2183 | -0.5398 to 0.1032 | No | ns | 0.1024 |
| CL4 vs. CL4-B1 | -1.146 | -2.111 to -0.1816 | Yes | \* | 0.0359 |
| B1 vs. CL4-B1 | -0.9278 | -1.769 to -0.08647 | Yes | \* | 0.0401 |
| | | | | | |
| Row 8 | | | | | |
| CL4 vs. B1 | 1.066 | 0.4132 to 1.719 | Yes | \* | 0.0111 |
| CL4 vs. CL4-B1 | 0.7958 | 0.07877 to 1.513 | Yes | \* | 0.0359 |
| B1 vs. CL4-B1 | -0.2704 | -0.9237 to 0.3830 | No | ns | 0.3642 |
Table S6. ANOVA and Tukey’s test tables for isoValeric acid production in CL4-B1 mono- and co-cultures

## Slide 8
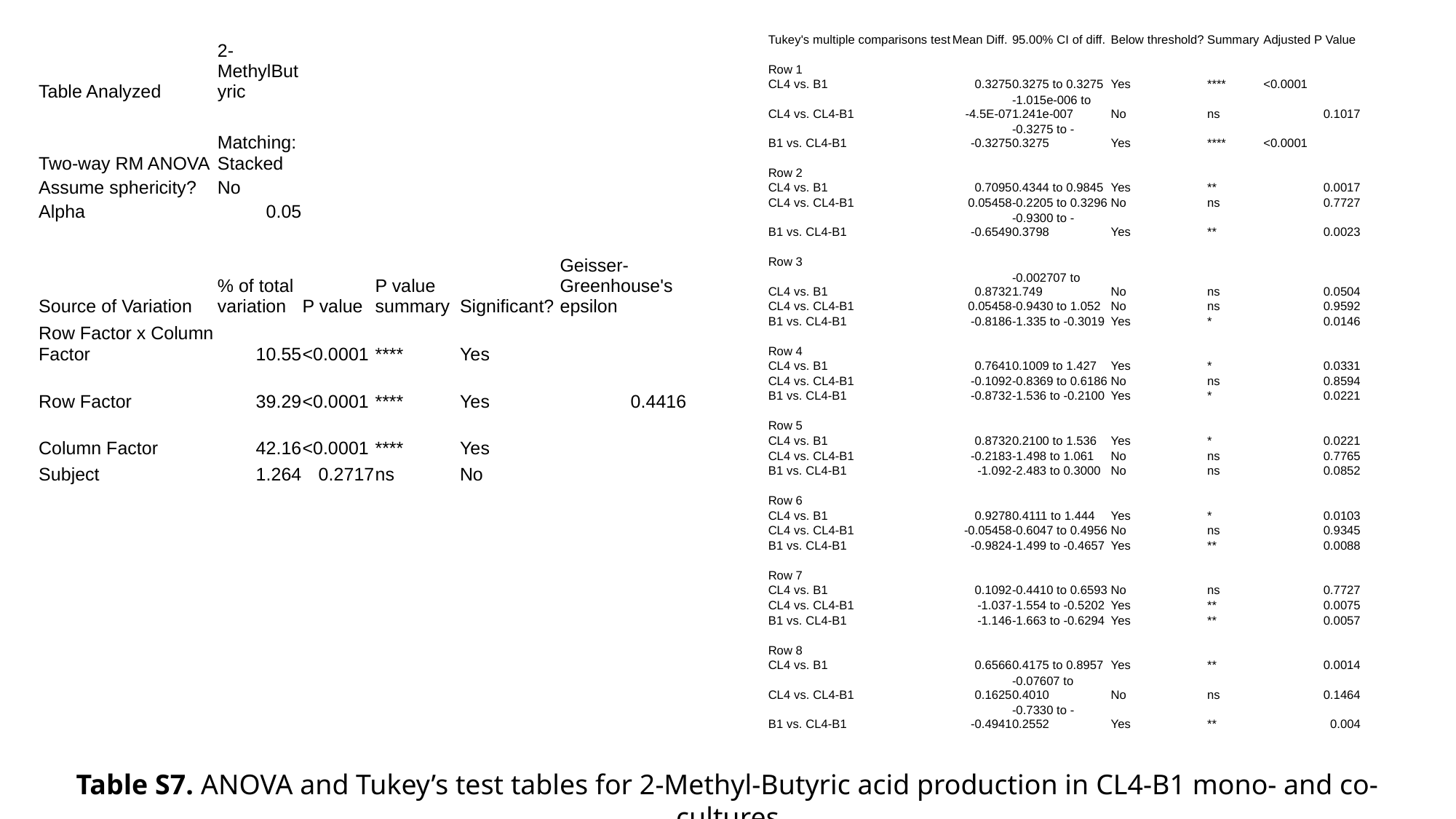

| Table Analyzed | 2-MethylButyric | | | | |
| --- | --- | --- | --- | --- | --- |
| | | | | | |
| Two-way RM ANOVA | Matching: Stacked | | | | |
| Assume sphericity? | No | | | | |
| Alpha | 0.05 | | | | |
| | | | | | |
| Source of Variation | % of total variation | P value | P value summary | Significant? | Geisser-Greenhouse's epsilon |
| Row Factor x Column Factor | 10.55 | <0.0001 | \*\*\*\* | Yes | |
| Row Factor | 39.29 | <0.0001 | \*\*\*\* | Yes | 0.4416 |
| Column Factor | 42.16 | <0.0001 | \*\*\*\* | Yes | |
| Subject | 1.264 | 0.2717 | ns | No | |
| Tukey's multiple comparisons test | Mean Diff. | 95.00% CI of diff. | Below threshold? | Summary | Adjusted P Value |
| --- | --- | --- | --- | --- | --- |
| | | | | | |
| Row 1 | | | | | |
| CL4 vs. B1 | 0.3275 | 0.3275 to 0.3275 | Yes | \*\*\*\* | <0.0001 |
| CL4 vs. CL4-B1 | -4.5E-07 | -1.015e-006 to 1.241e-007 | No | ns | 0.1017 |
| B1 vs. CL4-B1 | -0.3275 | -0.3275 to -0.3275 | Yes | \*\*\*\* | <0.0001 |
| | | | | | |
| Row 2 | | | | | |
| CL4 vs. B1 | 0.7095 | 0.4344 to 0.9845 | Yes | \*\* | 0.0017 |
| CL4 vs. CL4-B1 | 0.05458 | -0.2205 to 0.3296 | No | ns | 0.7727 |
| B1 vs. CL4-B1 | -0.6549 | -0.9300 to -0.3798 | Yes | \*\* | 0.0023 |
| | | | | | |
| Row 3 | | | | | |
| CL4 vs. B1 | 0.8732 | -0.002707 to 1.749 | No | ns | 0.0504 |
| CL4 vs. CL4-B1 | 0.05458 | -0.9430 to 1.052 | No | ns | 0.9592 |
| B1 vs. CL4-B1 | -0.8186 | -1.335 to -0.3019 | Yes | \* | 0.0146 |
| | | | | | |
| Row 4 | | | | | |
| CL4 vs. B1 | 0.7641 | 0.1009 to 1.427 | Yes | \* | 0.0331 |
| CL4 vs. CL4-B1 | -0.1092 | -0.8369 to 0.6186 | No | ns | 0.8594 |
| B1 vs. CL4-B1 | -0.8732 | -1.536 to -0.2100 | Yes | \* | 0.0221 |
| | | | | | |
| Row 5 | | | | | |
| CL4 vs. B1 | 0.8732 | 0.2100 to 1.536 | Yes | \* | 0.0221 |
| CL4 vs. CL4-B1 | -0.2183 | -1.498 to 1.061 | No | ns | 0.7765 |
| B1 vs. CL4-B1 | -1.092 | -2.483 to 0.3000 | No | ns | 0.0852 |
| | | | | | |
| Row 6 | | | | | |
| CL4 vs. B1 | 0.9278 | 0.4111 to 1.444 | Yes | \* | 0.0103 |
| CL4 vs. CL4-B1 | -0.05458 | -0.6047 to 0.4956 | No | ns | 0.9345 |
| B1 vs. CL4-B1 | -0.9824 | -1.499 to -0.4657 | Yes | \*\* | 0.0088 |
| | | | | | |
| Row 7 | | | | | |
| CL4 vs. B1 | 0.1092 | -0.4410 to 0.6593 | No | ns | 0.7727 |
| CL4 vs. CL4-B1 | -1.037 | -1.554 to -0.5202 | Yes | \*\* | 0.0075 |
| B1 vs. CL4-B1 | -1.146 | -1.663 to -0.6294 | Yes | \*\* | 0.0057 |
| | | | | | |
| Row 8 | | | | | |
| CL4 vs. B1 | 0.6566 | 0.4175 to 0.8957 | Yes | \*\* | 0.0014 |
| CL4 vs. CL4-B1 | 0.1625 | -0.07607 to 0.4010 | No | ns | 0.1464 |
| B1 vs. CL4-B1 | -0.4941 | -0.7330 to -0.2552 | Yes | \*\* | 0.004 |
Table S7. ANOVA and Tukey’s test tables for 2-Methyl-Butyric acid production in CL4-B1 mono- and co-cultures

## Slide 9
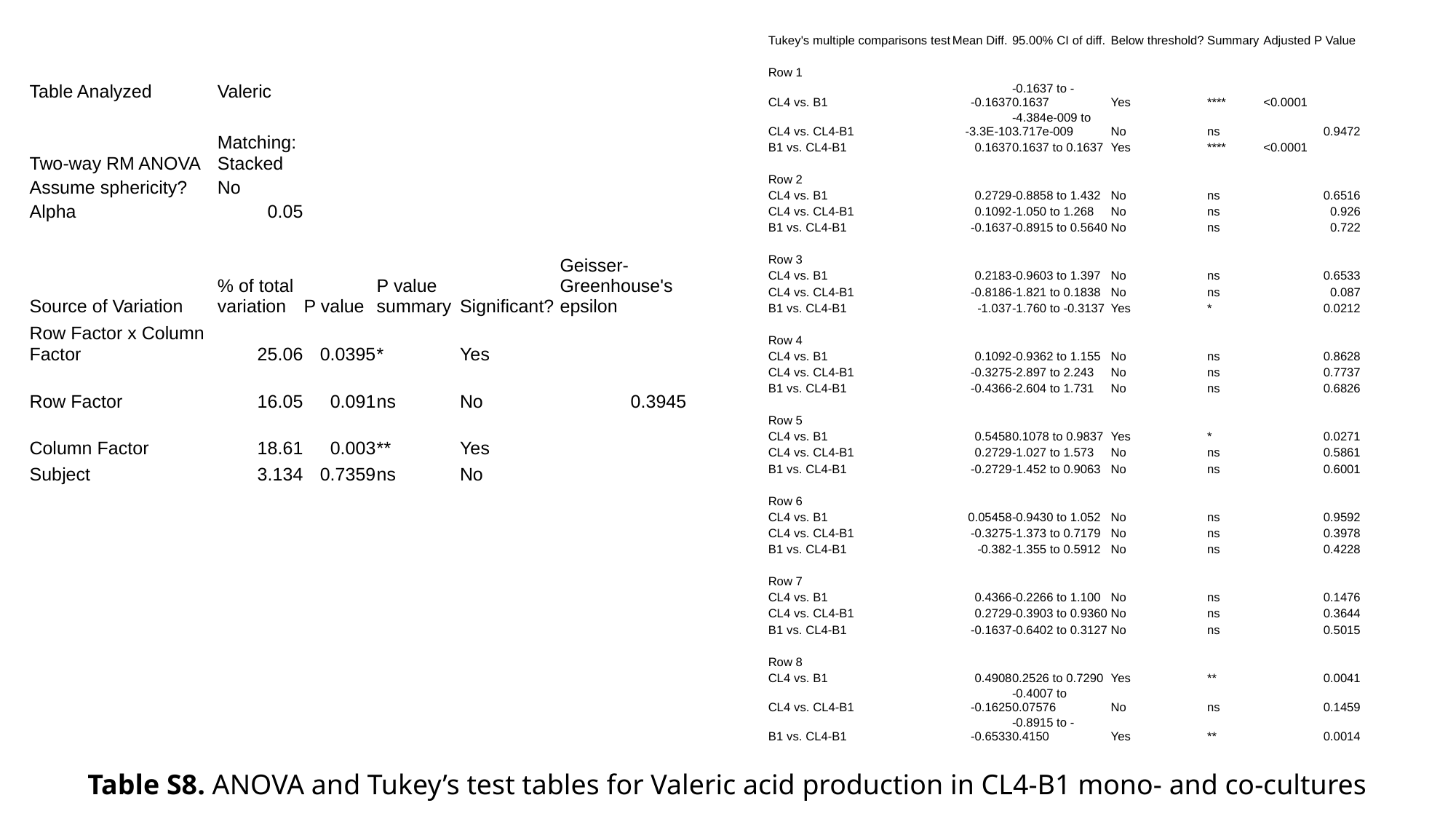

| Table Analyzed | Valeric | | | | |
| --- | --- | --- | --- | --- | --- |
| | | | | | |
| Two-way RM ANOVA | Matching: Stacked | | | | |
| Assume sphericity? | No | | | | |
| Alpha | 0.05 | | | | |
| | | | | | |
| Source of Variation | % of total variation | P value | P value summary | Significant? | Geisser-Greenhouse's epsilon |
| Row Factor x Column Factor | 25.06 | 0.0395 | \* | Yes | |
| Row Factor | 16.05 | 0.091 | ns | No | 0.3945 |
| Column Factor | 18.61 | 0.003 | \*\* | Yes | |
| Subject | 3.134 | 0.7359 | ns | No | |
| Tukey's multiple comparisons test | Mean Diff. | 95.00% CI of diff. | Below threshold? | Summary | Adjusted P Value |
| --- | --- | --- | --- | --- | --- |
| | | | | | |
| Row 1 | | | | | |
| CL4 vs. B1 | -0.1637 | -0.1637 to -0.1637 | Yes | \*\*\*\* | <0.0001 |
| CL4 vs. CL4-B1 | -3.3E-10 | -4.384e-009 to 3.717e-009 | No | ns | 0.9472 |
| B1 vs. CL4-B1 | 0.1637 | 0.1637 to 0.1637 | Yes | \*\*\*\* | <0.0001 |
| | | | | | |
| Row 2 | | | | | |
| CL4 vs. B1 | 0.2729 | -0.8858 to 1.432 | No | ns | 0.6516 |
| CL4 vs. CL4-B1 | 0.1092 | -1.050 to 1.268 | No | ns | 0.926 |
| B1 vs. CL4-B1 | -0.1637 | -0.8915 to 0.5640 | No | ns | 0.722 |
| | | | | | |
| Row 3 | | | | | |
| CL4 vs. B1 | 0.2183 | -0.9603 to 1.397 | No | ns | 0.6533 |
| CL4 vs. CL4-B1 | -0.8186 | -1.821 to 0.1838 | No | ns | 0.087 |
| B1 vs. CL4-B1 | -1.037 | -1.760 to -0.3137 | Yes | \* | 0.0212 |
| | | | | | |
| Row 4 | | | | | |
| CL4 vs. B1 | 0.1092 | -0.9362 to 1.155 | No | ns | 0.8628 |
| CL4 vs. CL4-B1 | -0.3275 | -2.897 to 2.243 | No | ns | 0.7737 |
| B1 vs. CL4-B1 | -0.4366 | -2.604 to 1.731 | No | ns | 0.6826 |
| | | | | | |
| Row 5 | | | | | |
| CL4 vs. B1 | 0.5458 | 0.1078 to 0.9837 | Yes | \* | 0.0271 |
| CL4 vs. CL4-B1 | 0.2729 | -1.027 to 1.573 | No | ns | 0.5861 |
| B1 vs. CL4-B1 | -0.2729 | -1.452 to 0.9063 | No | ns | 0.6001 |
| | | | | | |
| Row 6 | | | | | |
| CL4 vs. B1 | 0.05458 | -0.9430 to 1.052 | No | ns | 0.9592 |
| CL4 vs. CL4-B1 | -0.3275 | -1.373 to 0.7179 | No | ns | 0.3978 |
| B1 vs. CL4-B1 | -0.382 | -1.355 to 0.5912 | No | ns | 0.4228 |
| | | | | | |
| Row 7 | | | | | |
| CL4 vs. B1 | 0.4366 | -0.2266 to 1.100 | No | ns | 0.1476 |
| CL4 vs. CL4-B1 | 0.2729 | -0.3903 to 0.9360 | No | ns | 0.3644 |
| B1 vs. CL4-B1 | -0.1637 | -0.6402 to 0.3127 | No | ns | 0.5015 |
| | | | | | |
| Row 8 | | | | | |
| CL4 vs. B1 | 0.4908 | 0.2526 to 0.7290 | Yes | \*\* | 0.0041 |
| CL4 vs. CL4-B1 | -0.1625 | -0.4007 to 0.07576 | No | ns | 0.1459 |
| B1 vs. CL4-B1 | -0.6533 | -0.8915 to -0.4150 | Yes | \*\* | 0.0014 |
Table S8. ANOVA and Tukey’s test tables for Valeric acid production in CL4-B1 mono- and co-cultures

## Slide 10
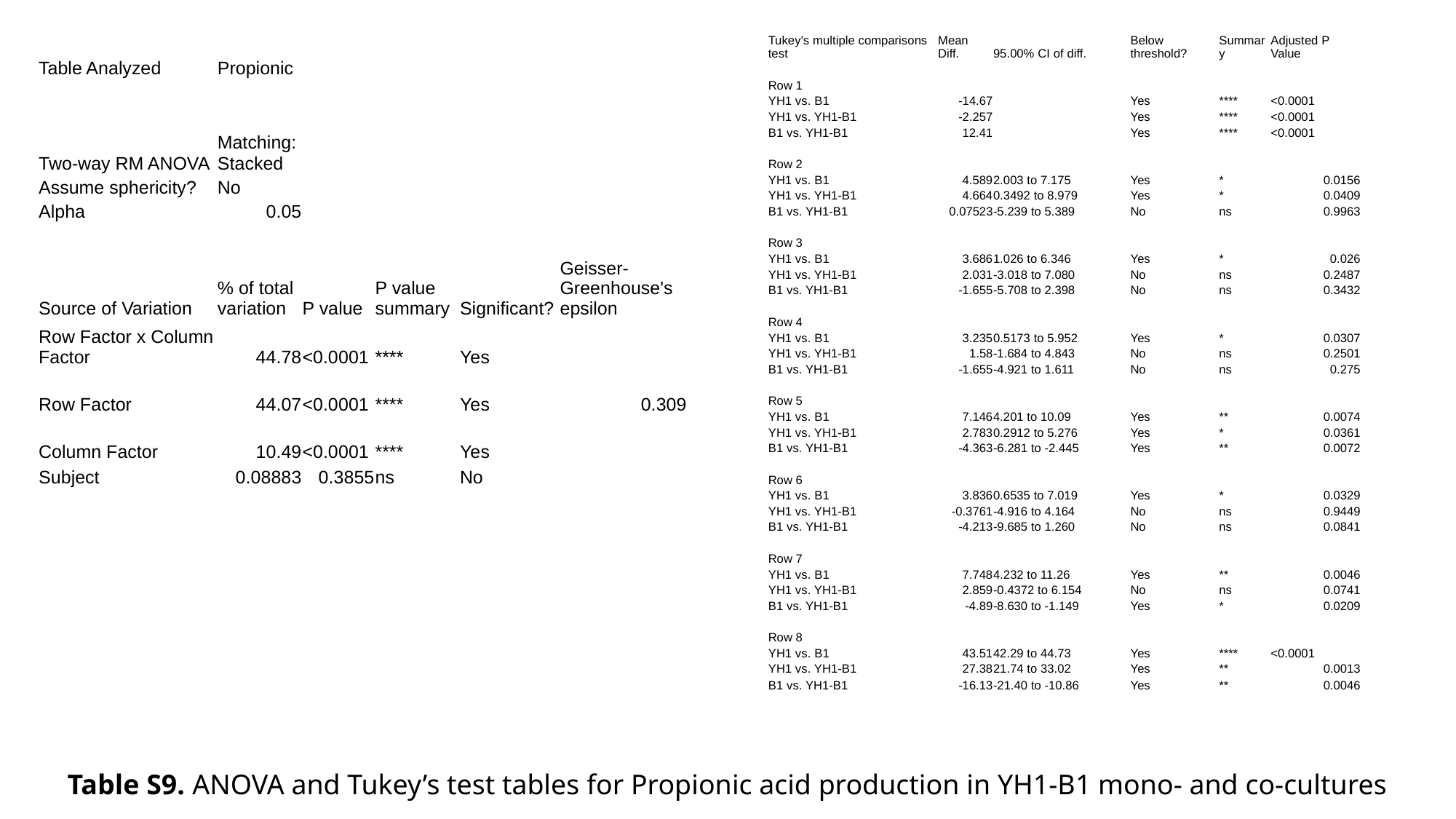

| Table Analyzed | Propionic | | | | |
| --- | --- | --- | --- | --- | --- |
| | | | | | |
| Two-way RM ANOVA | Matching: Stacked | | | | |
| Assume sphericity? | No | | | | |
| Alpha | 0.05 | | | | |
| | | | | | |
| Source of Variation | % of total variation | P value | P value summary | Significant? | Geisser-Greenhouse's epsilon |
| Row Factor x Column Factor | 44.78 | <0.0001 | \*\*\*\* | Yes | |
| Row Factor | 44.07 | <0.0001 | \*\*\*\* | Yes | 0.309 |
| Column Factor | 10.49 | <0.0001 | \*\*\*\* | Yes | |
| Subject | 0.08883 | 0.3855 | ns | No | |
| Tukey's multiple comparisons test | Mean Diff. | 95.00% CI of diff. | Below threshold? | Summary | Adjusted P Value |
| --- | --- | --- | --- | --- | --- |
| | | | | | |
| Row 1 | | | | | |
| YH1 vs. B1 | -14.67 | | Yes | \*\*\*\* | <0.0001 |
| YH1 vs. YH1-B1 | -2.257 | | Yes | \*\*\*\* | <0.0001 |
| B1 vs. YH1-B1 | 12.41 | | Yes | \*\*\*\* | <0.0001 |
| | | | | | |
| Row 2 | | | | | |
| YH1 vs. B1 | 4.589 | 2.003 to 7.175 | Yes | \* | 0.0156 |
| YH1 vs. YH1-B1 | 4.664 | 0.3492 to 8.979 | Yes | \* | 0.0409 |
| B1 vs. YH1-B1 | 0.07523 | -5.239 to 5.389 | No | ns | 0.9963 |
| | | | | | |
| Row 3 | | | | | |
| YH1 vs. B1 | 3.686 | 1.026 to 6.346 | Yes | \* | 0.026 |
| YH1 vs. YH1-B1 | 2.031 | -3.018 to 7.080 | No | ns | 0.2487 |
| B1 vs. YH1-B1 | -1.655 | -5.708 to 2.398 | No | ns | 0.3432 |
| | | | | | |
| Row 4 | | | | | |
| YH1 vs. B1 | 3.235 | 0.5173 to 5.952 | Yes | \* | 0.0307 |
| YH1 vs. YH1-B1 | 1.58 | -1.684 to 4.843 | No | ns | 0.2501 |
| B1 vs. YH1-B1 | -1.655 | -4.921 to 1.611 | No | ns | 0.275 |
| | | | | | |
| Row 5 | | | | | |
| YH1 vs. B1 | 7.146 | 4.201 to 10.09 | Yes | \*\* | 0.0074 |
| YH1 vs. YH1-B1 | 2.783 | 0.2912 to 5.276 | Yes | \* | 0.0361 |
| B1 vs. YH1-B1 | -4.363 | -6.281 to -2.445 | Yes | \*\* | 0.0072 |
| | | | | | |
| Row 6 | | | | | |
| YH1 vs. B1 | 3.836 | 0.6535 to 7.019 | Yes | \* | 0.0329 |
| YH1 vs. YH1-B1 | -0.3761 | -4.916 to 4.164 | No | ns | 0.9449 |
| B1 vs. YH1-B1 | -4.213 | -9.685 to 1.260 | No | ns | 0.0841 |
| | | | | | |
| Row 7 | | | | | |
| YH1 vs. B1 | 7.748 | 4.232 to 11.26 | Yes | \*\* | 0.0046 |
| YH1 vs. YH1-B1 | 2.859 | -0.4372 to 6.154 | No | ns | 0.0741 |
| B1 vs. YH1-B1 | -4.89 | -8.630 to -1.149 | Yes | \* | 0.0209 |
| | | | | | |
| Row 8 | | | | | |
| YH1 vs. B1 | 43.51 | 42.29 to 44.73 | Yes | \*\*\*\* | <0.0001 |
| YH1 vs. YH1-B1 | 27.38 | 21.74 to 33.02 | Yes | \*\* | 0.0013 |
| B1 vs. YH1-B1 | -16.13 | -21.40 to -10.86 | Yes | \*\* | 0.0046 |
Table S9. ANOVA and Tukey’s test tables for Propionic acid production in YH1-B1 mono- and co-cultures

## Slide 11
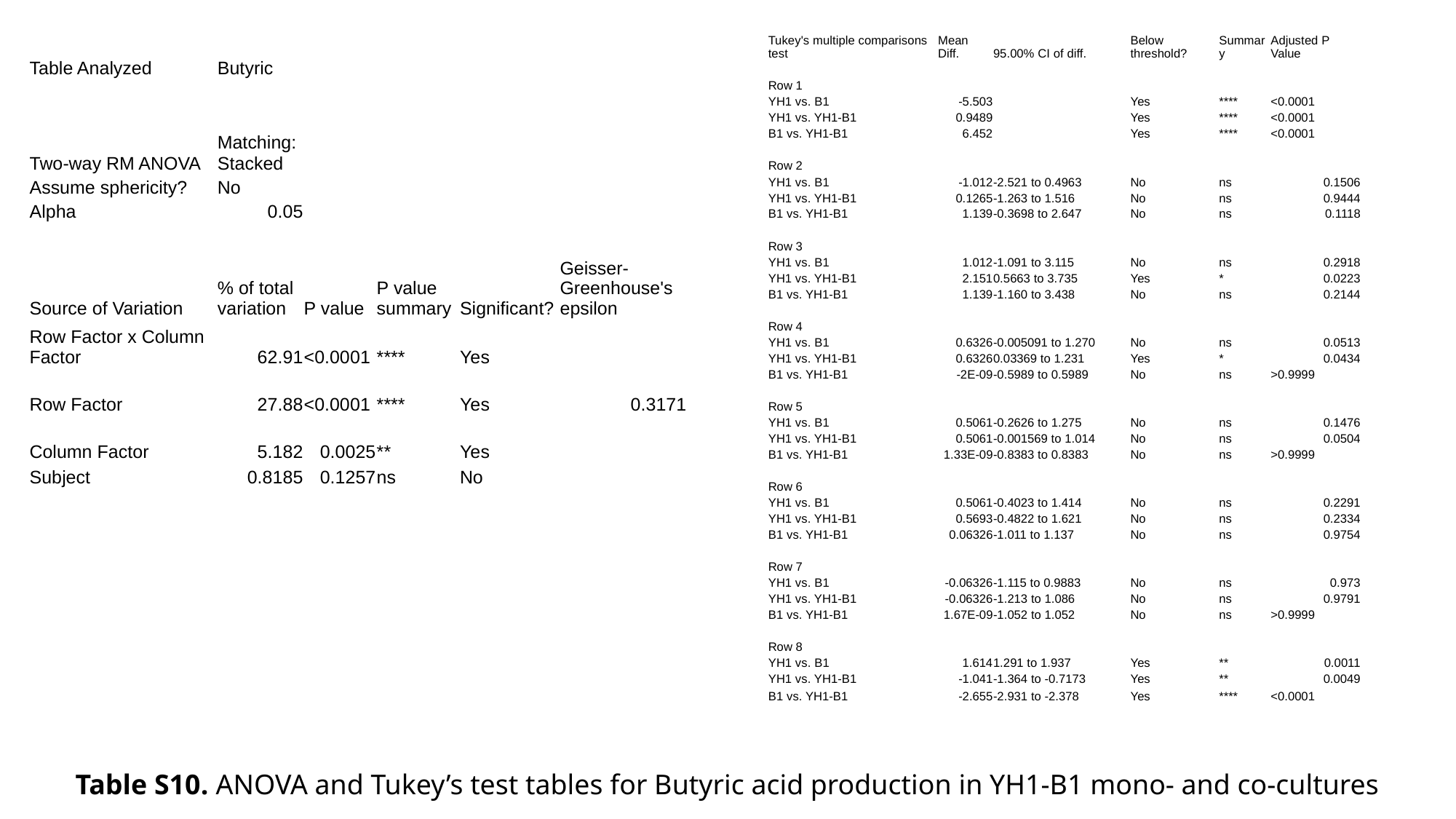

| Table Analyzed | Butyric | | | | |
| --- | --- | --- | --- | --- | --- |
| | | | | | |
| Two-way RM ANOVA | Matching: Stacked | | | | |
| Assume sphericity? | No | | | | |
| Alpha | 0.05 | | | | |
| | | | | | |
| Source of Variation | % of total variation | P value | P value summary | Significant? | Geisser-Greenhouse's epsilon |
| Row Factor x Column Factor | 62.91 | <0.0001 | \*\*\*\* | Yes | |
| Row Factor | 27.88 | <0.0001 | \*\*\*\* | Yes | 0.3171 |
| Column Factor | 5.182 | 0.0025 | \*\* | Yes | |
| Subject | 0.8185 | 0.1257 | ns | No | |
| Tukey's multiple comparisons test | Mean Diff. | 95.00% CI of diff. | Below threshold? | Summary | Adjusted P Value |
| --- | --- | --- | --- | --- | --- |
| | | | | | |
| Row 1 | | | | | |
| YH1 vs. B1 | -5.503 | | Yes | \*\*\*\* | <0.0001 |
| YH1 vs. YH1-B1 | 0.9489 | | Yes | \*\*\*\* | <0.0001 |
| B1 vs. YH1-B1 | 6.452 | | Yes | \*\*\*\* | <0.0001 |
| | | | | | |
| Row 2 | | | | | |
| YH1 vs. B1 | -1.012 | -2.521 to 0.4963 | No | ns | 0.1506 |
| YH1 vs. YH1-B1 | 0.1265 | -1.263 to 1.516 | No | ns | 0.9444 |
| B1 vs. YH1-B1 | 1.139 | -0.3698 to 2.647 | No | ns | 0.1118 |
| | | | | | |
| Row 3 | | | | | |
| YH1 vs. B1 | 1.012 | -1.091 to 3.115 | No | ns | 0.2918 |
| YH1 vs. YH1-B1 | 2.151 | 0.5663 to 3.735 | Yes | \* | 0.0223 |
| B1 vs. YH1-B1 | 1.139 | -1.160 to 3.438 | No | ns | 0.2144 |
| | | | | | |
| Row 4 | | | | | |
| YH1 vs. B1 | 0.6326 | -0.005091 to 1.270 | No | ns | 0.0513 |
| YH1 vs. YH1-B1 | 0.6326 | 0.03369 to 1.231 | Yes | \* | 0.0434 |
| B1 vs. YH1-B1 | -2E-09 | -0.5989 to 0.5989 | No | ns | >0.9999 |
| | | | | | |
| Row 5 | | | | | |
| YH1 vs. B1 | 0.5061 | -0.2626 to 1.275 | No | ns | 0.1476 |
| YH1 vs. YH1-B1 | 0.5061 | -0.001569 to 1.014 | No | ns | 0.0504 |
| B1 vs. YH1-B1 | 1.33E-09 | -0.8383 to 0.8383 | No | ns | >0.9999 |
| | | | | | |
| Row 6 | | | | | |
| YH1 vs. B1 | 0.5061 | -0.4023 to 1.414 | No | ns | 0.2291 |
| YH1 vs. YH1-B1 | 0.5693 | -0.4822 to 1.621 | No | ns | 0.2334 |
| B1 vs. YH1-B1 | 0.06326 | -1.011 to 1.137 | No | ns | 0.9754 |
| | | | | | |
| Row 7 | | | | | |
| YH1 vs. B1 | -0.06326 | -1.115 to 0.9883 | No | ns | 0.973 |
| YH1 vs. YH1-B1 | -0.06326 | -1.213 to 1.086 | No | ns | 0.9791 |
| B1 vs. YH1-B1 | 1.67E-09 | -1.052 to 1.052 | No | ns | >0.9999 |
| | | | | | |
| Row 8 | | | | | |
| YH1 vs. B1 | 1.614 | 1.291 to 1.937 | Yes | \*\* | 0.0011 |
| YH1 vs. YH1-B1 | -1.041 | -1.364 to -0.7173 | Yes | \*\* | 0.0049 |
| B1 vs. YH1-B1 | -2.655 | -2.931 to -2.378 | Yes | \*\*\*\* | <0.0001 |
Table S10. ANOVA and Tukey’s test tables for Butyric acid production in YH1-B1 mono- and co-cultures

## Slide 12
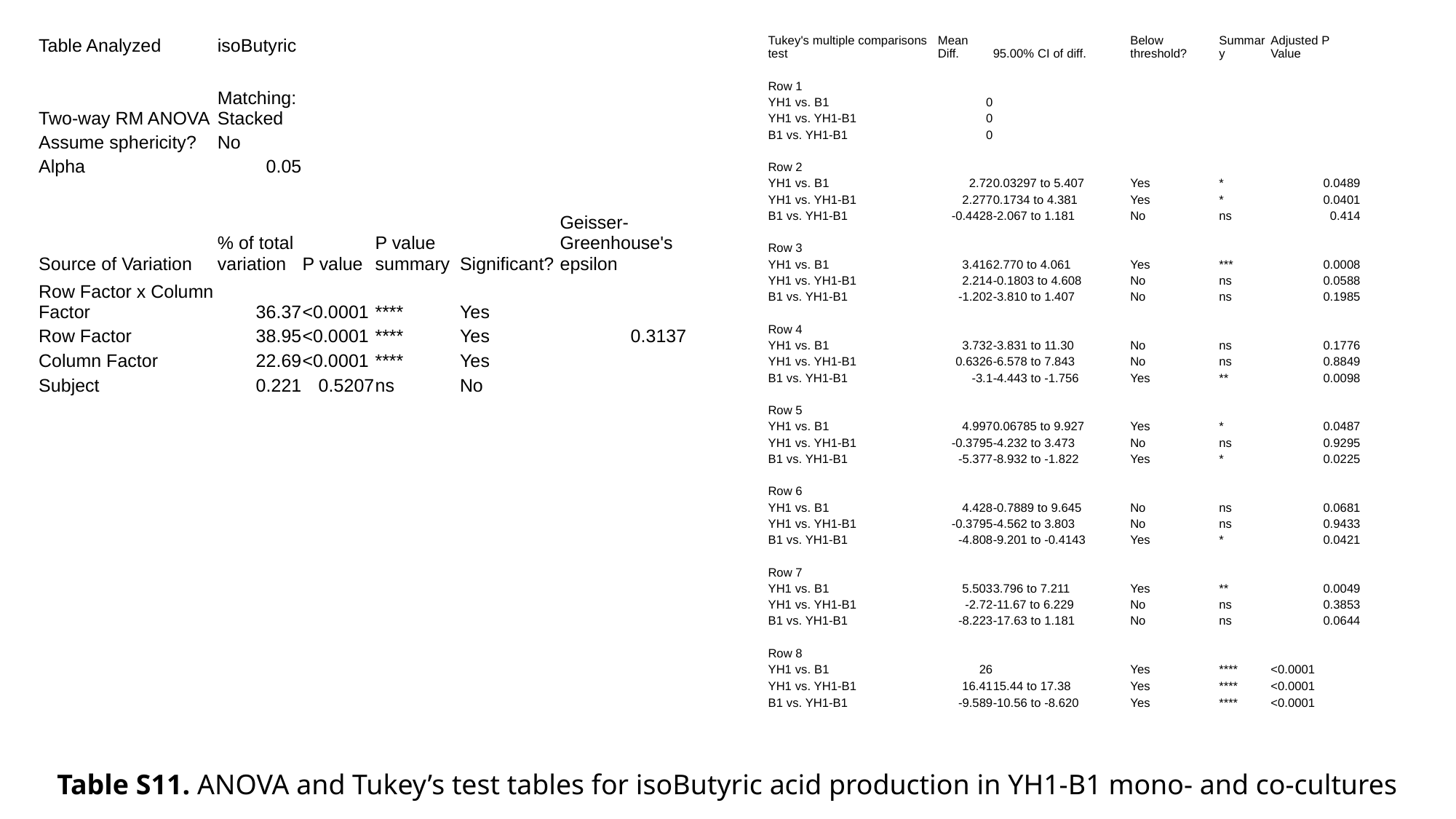

| Table Analyzed | isoButyric | | | | |
| --- | --- | --- | --- | --- | --- |
| | | | | | |
| Two-way RM ANOVA | Matching: Stacked | | | | |
| Assume sphericity? | No | | | | |
| Alpha | 0.05 | | | | |
| | | | | | |
| Source of Variation | % of total variation | P value | P value summary | Significant? | Geisser-Greenhouse's epsilon |
| Row Factor x Column Factor | 36.37 | <0.0001 | \*\*\*\* | Yes | |
| Row Factor | 38.95 | <0.0001 | \*\*\*\* | Yes | 0.3137 |
| Column Factor | 22.69 | <0.0001 | \*\*\*\* | Yes | |
| Subject | 0.221 | 0.5207 | ns | No | |
| Tukey's multiple comparisons test | Mean Diff. | 95.00% CI of diff. | Below threshold? | Summary | Adjusted P Value |
| --- | --- | --- | --- | --- | --- |
| | | | | | |
| Row 1 | | | | | |
| YH1 vs. B1 | 0 | | | | |
| YH1 vs. YH1-B1 | 0 | | | | |
| B1 vs. YH1-B1 | 0 | | | | |
| | | | | | |
| Row 2 | | | | | |
| YH1 vs. B1 | 2.72 | 0.03297 to 5.407 | Yes | \* | 0.0489 |
| YH1 vs. YH1-B1 | 2.277 | 0.1734 to 4.381 | Yes | \* | 0.0401 |
| B1 vs. YH1-B1 | -0.4428 | -2.067 to 1.181 | No | ns | 0.414 |
| | | | | | |
| Row 3 | | | | | |
| YH1 vs. B1 | 3.416 | 2.770 to 4.061 | Yes | \*\*\* | 0.0008 |
| YH1 vs. YH1-B1 | 2.214 | -0.1803 to 4.608 | No | ns | 0.0588 |
| B1 vs. YH1-B1 | -1.202 | -3.810 to 1.407 | No | ns | 0.1985 |
| | | | | | |
| Row 4 | | | | | |
| YH1 vs. B1 | 3.732 | -3.831 to 11.30 | No | ns | 0.1776 |
| YH1 vs. YH1-B1 | 0.6326 | -6.578 to 7.843 | No | ns | 0.8849 |
| B1 vs. YH1-B1 | -3.1 | -4.443 to -1.756 | Yes | \*\* | 0.0098 |
| | | | | | |
| Row 5 | | | | | |
| YH1 vs. B1 | 4.997 | 0.06785 to 9.927 | Yes | \* | 0.0487 |
| YH1 vs. YH1-B1 | -0.3795 | -4.232 to 3.473 | No | ns | 0.9295 |
| B1 vs. YH1-B1 | -5.377 | -8.932 to -1.822 | Yes | \* | 0.0225 |
| | | | | | |
| Row 6 | | | | | |
| YH1 vs. B1 | 4.428 | -0.7889 to 9.645 | No | ns | 0.0681 |
| YH1 vs. YH1-B1 | -0.3795 | -4.562 to 3.803 | No | ns | 0.9433 |
| B1 vs. YH1-B1 | -4.808 | -9.201 to -0.4143 | Yes | \* | 0.0421 |
| | | | | | |
| Row 7 | | | | | |
| YH1 vs. B1 | 5.503 | 3.796 to 7.211 | Yes | \*\* | 0.0049 |
| YH1 vs. YH1-B1 | -2.72 | -11.67 to 6.229 | No | ns | 0.3853 |
| B1 vs. YH1-B1 | -8.223 | -17.63 to 1.181 | No | ns | 0.0644 |
| | | | | | |
| Row 8 | | | | | |
| YH1 vs. B1 | 26 | | Yes | \*\*\*\* | <0.0001 |
| YH1 vs. YH1-B1 | 16.41 | 15.44 to 17.38 | Yes | \*\*\*\* | <0.0001 |
| B1 vs. YH1-B1 | -9.589 | -10.56 to -8.620 | Yes | \*\*\*\* | <0.0001 |
Table S11. ANOVA and Tukey’s test tables for isoButyric acid production in YH1-B1 mono- and co-cultures

## Slide 13
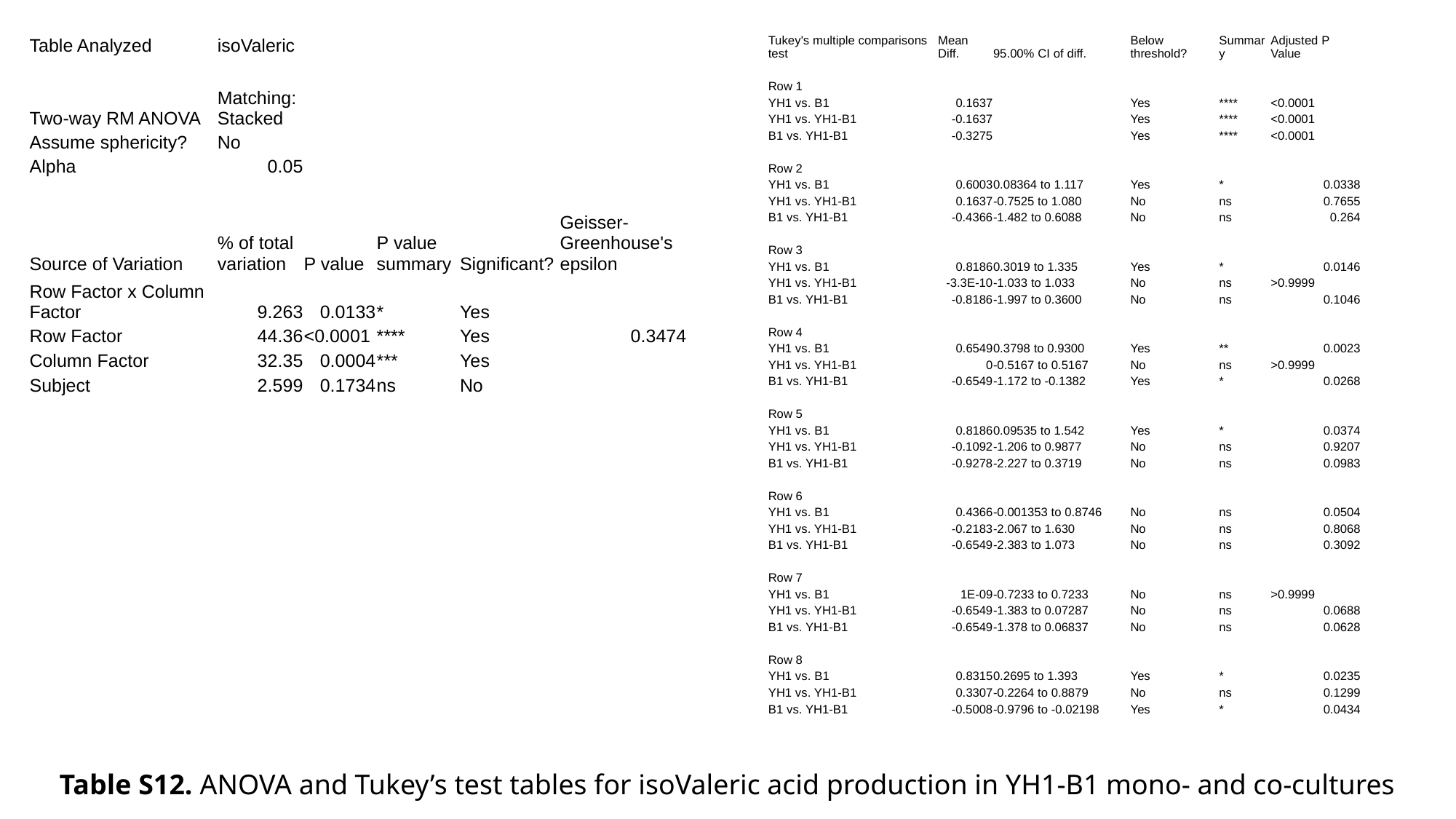

| Table Analyzed | isoValeric | | | | |
| --- | --- | --- | --- | --- | --- |
| | | | | | |
| Two-way RM ANOVA | Matching: Stacked | | | | |
| Assume sphericity? | No | | | | |
| Alpha | 0.05 | | | | |
| | | | | | |
| Source of Variation | % of total variation | P value | P value summary | Significant? | Geisser-Greenhouse's epsilon |
| Row Factor x Column Factor | 9.263 | 0.0133 | \* | Yes | |
| Row Factor | 44.36 | <0.0001 | \*\*\*\* | Yes | 0.3474 |
| Column Factor | 32.35 | 0.0004 | \*\*\* | Yes | |
| Subject | 2.599 | 0.1734 | ns | No | |
| Tukey's multiple comparisons test | Mean Diff. | 95.00% CI of diff. | Below threshold? | Summary | Adjusted P Value |
| --- | --- | --- | --- | --- | --- |
| | | | | | |
| Row 1 | | | | | |
| YH1 vs. B1 | 0.1637 | | Yes | \*\*\*\* | <0.0001 |
| YH1 vs. YH1-B1 | -0.1637 | | Yes | \*\*\*\* | <0.0001 |
| B1 vs. YH1-B1 | -0.3275 | | Yes | \*\*\*\* | <0.0001 |
| | | | | | |
| Row 2 | | | | | |
| YH1 vs. B1 | 0.6003 | 0.08364 to 1.117 | Yes | \* | 0.0338 |
| YH1 vs. YH1-B1 | 0.1637 | -0.7525 to 1.080 | No | ns | 0.7655 |
| B1 vs. YH1-B1 | -0.4366 | -1.482 to 0.6088 | No | ns | 0.264 |
| | | | | | |
| Row 3 | | | | | |
| YH1 vs. B1 | 0.8186 | 0.3019 to 1.335 | Yes | \* | 0.0146 |
| YH1 vs. YH1-B1 | -3.3E-10 | -1.033 to 1.033 | No | ns | >0.9999 |
| B1 vs. YH1-B1 | -0.8186 | -1.997 to 0.3600 | No | ns | 0.1046 |
| | | | | | |
| Row 4 | | | | | |
| YH1 vs. B1 | 0.6549 | 0.3798 to 0.9300 | Yes | \*\* | 0.0023 |
| YH1 vs. YH1-B1 | 0 | -0.5167 to 0.5167 | No | ns | >0.9999 |
| B1 vs. YH1-B1 | -0.6549 | -1.172 to -0.1382 | Yes | \* | 0.0268 |
| | | | | | |
| Row 5 | | | | | |
| YH1 vs. B1 | 0.8186 | 0.09535 to 1.542 | Yes | \* | 0.0374 |
| YH1 vs. YH1-B1 | -0.1092 | -1.206 to 0.9877 | No | ns | 0.9207 |
| B1 vs. YH1-B1 | -0.9278 | -2.227 to 0.3719 | No | ns | 0.0983 |
| | | | | | |
| Row 6 | | | | | |
| YH1 vs. B1 | 0.4366 | -0.001353 to 0.8746 | No | ns | 0.0504 |
| YH1 vs. YH1-B1 | -0.2183 | -2.067 to 1.630 | No | ns | 0.8068 |
| B1 vs. YH1-B1 | -0.6549 | -2.383 to 1.073 | No | ns | 0.3092 |
| | | | | | |
| Row 7 | | | | | |
| YH1 vs. B1 | 1E-09 | -0.7233 to 0.7233 | No | ns | >0.9999 |
| YH1 vs. YH1-B1 | -0.6549 | -1.383 to 0.07287 | No | ns | 0.0688 |
| B1 vs. YH1-B1 | -0.6549 | -1.378 to 0.06837 | No | ns | 0.0628 |
| | | | | | |
| Row 8 | | | | | |
| YH1 vs. B1 | 0.8315 | 0.2695 to 1.393 | Yes | \* | 0.0235 |
| YH1 vs. YH1-B1 | 0.3307 | -0.2264 to 0.8879 | No | ns | 0.1299 |
| B1 vs. YH1-B1 | -0.5008 | -0.9796 to -0.02198 | Yes | \* | 0.0434 |
Table S12. ANOVA and Tukey’s test tables for isoValeric acid production in YH1-B1 mono- and co-cultures

## Slide 14
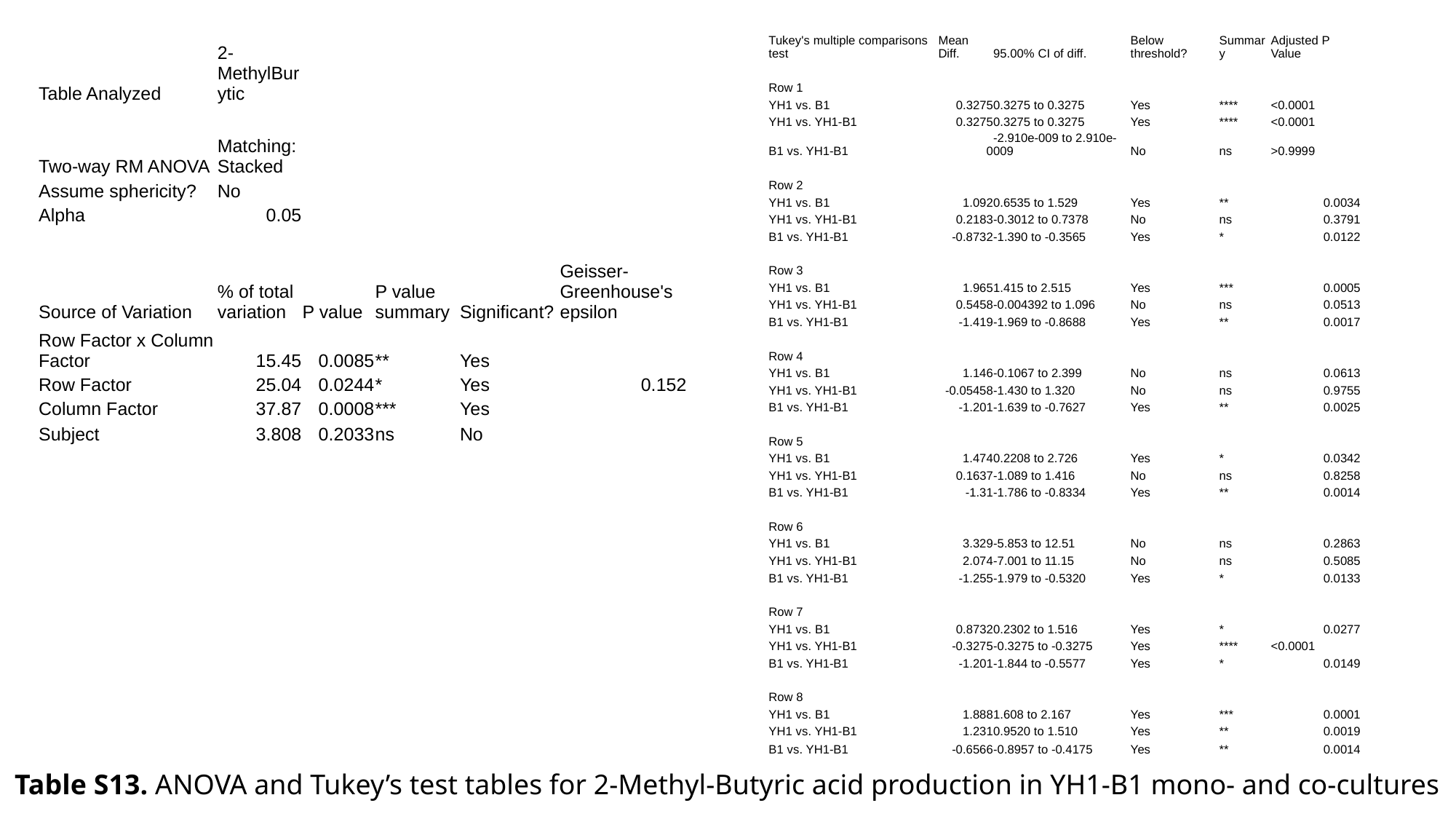

| Table Analyzed | 2-MethylBurytic | | | | |
| --- | --- | --- | --- | --- | --- |
| | | | | | |
| Two-way RM ANOVA | Matching: Stacked | | | | |
| Assume sphericity? | No | | | | |
| Alpha | 0.05 | | | | |
| | | | | | |
| Source of Variation | % of total variation | P value | P value summary | Significant? | Geisser-Greenhouse's epsilon |
| Row Factor x Column Factor | 15.45 | 0.0085 | \*\* | Yes | |
| Row Factor | 25.04 | 0.0244 | \* | Yes | 0.152 |
| Column Factor | 37.87 | 0.0008 | \*\*\* | Yes | |
| Subject | 3.808 | 0.2033 | ns | No | |
| Tukey's multiple comparisons test | Mean Diff. | 95.00% CI of diff. | Below threshold? | Summary | Adjusted P Value |
| --- | --- | --- | --- | --- | --- |
| | | | | | |
| Row 1 | | | | | |
| YH1 vs. B1 | 0.3275 | 0.3275 to 0.3275 | Yes | \*\*\*\* | <0.0001 |
| YH1 vs. YH1-B1 | 0.3275 | 0.3275 to 0.3275 | Yes | \*\*\*\* | <0.0001 |
| B1 vs. YH1-B1 | 0 | -2.910e-009 to 2.910e-009 | No | ns | >0.9999 |
| | | | | | |
| Row 2 | | | | | |
| YH1 vs. B1 | 1.092 | 0.6535 to 1.529 | Yes | \*\* | 0.0034 |
| YH1 vs. YH1-B1 | 0.2183 | -0.3012 to 0.7378 | No | ns | 0.3791 |
| B1 vs. YH1-B1 | -0.8732 | -1.390 to -0.3565 | Yes | \* | 0.0122 |
| | | | | | |
| Row 3 | | | | | |
| YH1 vs. B1 | 1.965 | 1.415 to 2.515 | Yes | \*\*\* | 0.0005 |
| YH1 vs. YH1-B1 | 0.5458 | -0.004392 to 1.096 | No | ns | 0.0513 |
| B1 vs. YH1-B1 | -1.419 | -1.969 to -0.8688 | Yes | \*\* | 0.0017 |
| | | | | | |
| Row 4 | | | | | |
| YH1 vs. B1 | 1.146 | -0.1067 to 2.399 | No | ns | 0.0613 |
| YH1 vs. YH1-B1 | -0.05458 | -1.430 to 1.320 | No | ns | 0.9755 |
| B1 vs. YH1-B1 | -1.201 | -1.639 to -0.7627 | Yes | \*\* | 0.0025 |
| | | | | | |
| Row 5 | | | | | |
| YH1 vs. B1 | 1.474 | 0.2208 to 2.726 | Yes | \* | 0.0342 |
| YH1 vs. YH1-B1 | 0.1637 | -1.089 to 1.416 | No | ns | 0.8258 |
| B1 vs. YH1-B1 | -1.31 | -1.786 to -0.8334 | Yes | \*\* | 0.0014 |
| | | | | | |
| Row 6 | | | | | |
| YH1 vs. B1 | 3.329 | -5.853 to 12.51 | No | ns | 0.2863 |
| YH1 vs. YH1-B1 | 2.074 | -7.001 to 11.15 | No | ns | 0.5085 |
| B1 vs. YH1-B1 | -1.255 | -1.979 to -0.5320 | Yes | \* | 0.0133 |
| | | | | | |
| Row 7 | | | | | |
| YH1 vs. B1 | 0.8732 | 0.2302 to 1.516 | Yes | \* | 0.0277 |
| YH1 vs. YH1-B1 | -0.3275 | -0.3275 to -0.3275 | Yes | \*\*\*\* | <0.0001 |
| B1 vs. YH1-B1 | -1.201 | -1.844 to -0.5577 | Yes | \* | 0.0149 |
| | | | | | |
| Row 8 | | | | | |
| YH1 vs. B1 | 1.888 | 1.608 to 2.167 | Yes | \*\*\* | 0.0001 |
| YH1 vs. YH1-B1 | 1.231 | 0.9520 to 1.510 | Yes | \*\* | 0.0019 |
| B1 vs. YH1-B1 | -0.6566 | -0.8957 to -0.4175 | Yes | \*\* | 0.0014 |
Table S13. ANOVA and Tukey’s test tables for 2-Methyl-Butyric acid production in YH1-B1 mono- and co-cultures

## Slide 15
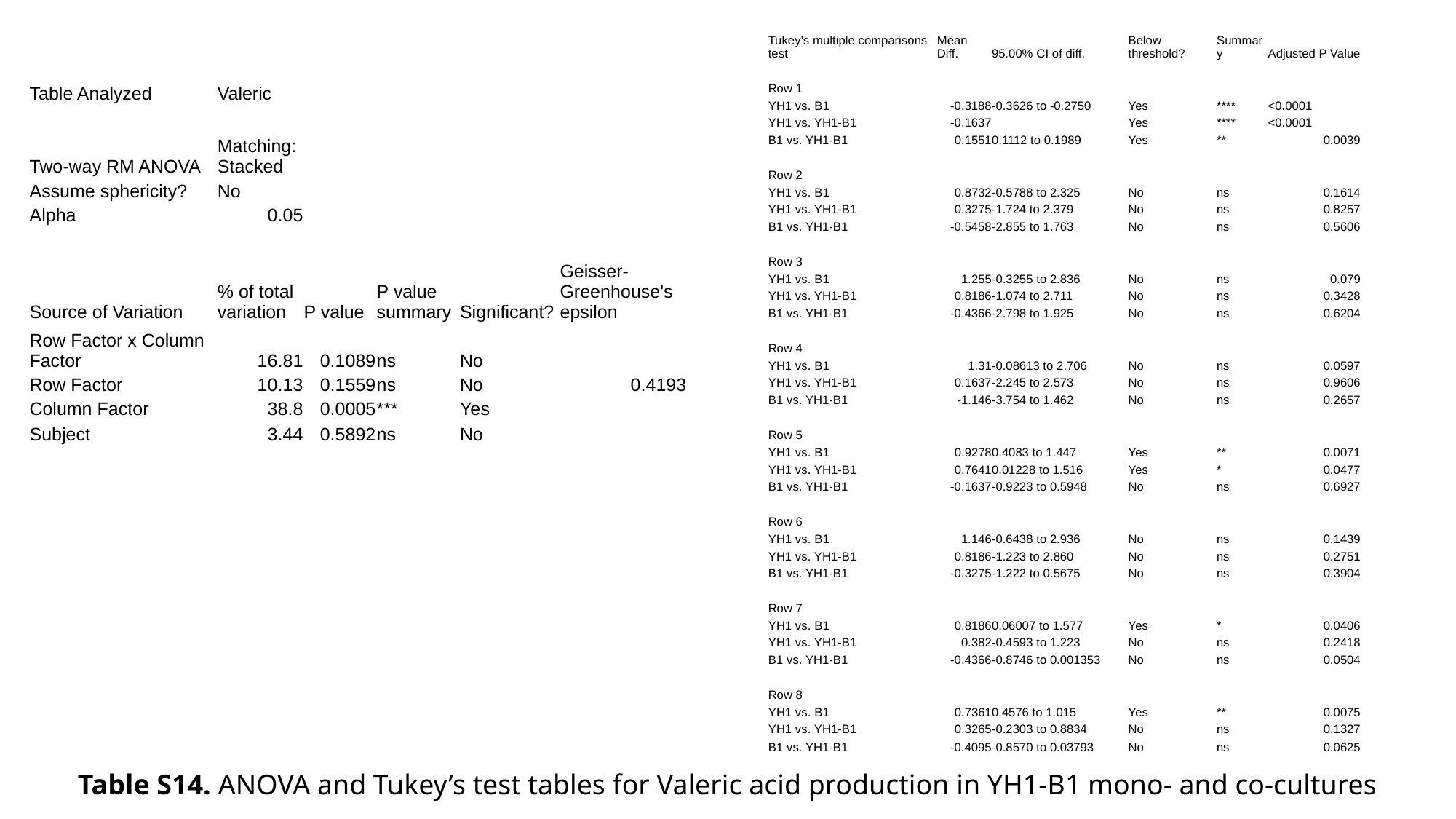

| Table Analyzed | Valeric | | | | |
| --- | --- | --- | --- | --- | --- |
| | | | | | |
| Two-way RM ANOVA | Matching: Stacked | | | | |
| Assume sphericity? | No | | | | |
| Alpha | 0.05 | | | | |
| | | | | | |
| Source of Variation | % of total variation | P value | P value summary | Significant? | Geisser-Greenhouse's epsilon |
| Row Factor x Column Factor | 16.81 | 0.1089 | ns | No | |
| Row Factor | 10.13 | 0.1559 | ns | No | 0.4193 |
| Column Factor | 38.8 | 0.0005 | \*\*\* | Yes | |
| Subject | 3.44 | 0.5892 | ns | No | |
| Tukey's multiple comparisons test | Mean Diff. | 95.00% CI of diff. | Below threshold? | Summary | Adjusted P Value |
| --- | --- | --- | --- | --- | --- |
| | | | | | |
| Row 1 | | | | | |
| YH1 vs. B1 | -0.3188 | -0.3626 to -0.2750 | Yes | \*\*\*\* | <0.0001 |
| YH1 vs. YH1-B1 | -0.1637 | | Yes | \*\*\*\* | <0.0001 |
| B1 vs. YH1-B1 | 0.1551 | 0.1112 to 0.1989 | Yes | \*\* | 0.0039 |
| | | | | | |
| Row 2 | | | | | |
| YH1 vs. B1 | 0.8732 | -0.5788 to 2.325 | No | ns | 0.1614 |
| YH1 vs. YH1-B1 | 0.3275 | -1.724 to 2.379 | No | ns | 0.8257 |
| B1 vs. YH1-B1 | -0.5458 | -2.855 to 1.763 | No | ns | 0.5606 |
| | | | | | |
| Row 3 | | | | | |
| YH1 vs. B1 | 1.255 | -0.3255 to 2.836 | No | ns | 0.079 |
| YH1 vs. YH1-B1 | 0.8186 | -1.074 to 2.711 | No | ns | 0.3428 |
| B1 vs. YH1-B1 | -0.4366 | -2.798 to 1.925 | No | ns | 0.6204 |
| | | | | | |
| Row 4 | | | | | |
| YH1 vs. B1 | 1.31 | -0.08613 to 2.706 | No | ns | 0.0597 |
| YH1 vs. YH1-B1 | 0.1637 | -2.245 to 2.573 | No | ns | 0.9606 |
| B1 vs. YH1-B1 | -1.146 | -3.754 to 1.462 | No | ns | 0.2657 |
| | | | | | |
| Row 5 | | | | | |
| YH1 vs. B1 | 0.9278 | 0.4083 to 1.447 | Yes | \*\* | 0.0071 |
| YH1 vs. YH1-B1 | 0.7641 | 0.01228 to 1.516 | Yes | \* | 0.0477 |
| B1 vs. YH1-B1 | -0.1637 | -0.9223 to 0.5948 | No | ns | 0.6927 |
| | | | | | |
| Row 6 | | | | | |
| YH1 vs. B1 | 1.146 | -0.6438 to 2.936 | No | ns | 0.1439 |
| YH1 vs. YH1-B1 | 0.8186 | -1.223 to 2.860 | No | ns | 0.2751 |
| B1 vs. YH1-B1 | -0.3275 | -1.222 to 0.5675 | No | ns | 0.3904 |
| | | | | | |
| Row 7 | | | | | |
| YH1 vs. B1 | 0.8186 | 0.06007 to 1.577 | Yes | \* | 0.0406 |
| YH1 vs. YH1-B1 | 0.382 | -0.4593 to 1.223 | No | ns | 0.2418 |
| B1 vs. YH1-B1 | -0.4366 | -0.8746 to 0.001353 | No | ns | 0.0504 |
| | | | | | |
| Row 8 | | | | | |
| YH1 vs. B1 | 0.7361 | 0.4576 to 1.015 | Yes | \*\* | 0.0075 |
| YH1 vs. YH1-B1 | 0.3265 | -0.2303 to 0.8834 | No | ns | 0.1327 |
| B1 vs. YH1-B1 | -0.4095 | -0.8570 to 0.03793 | No | ns | 0.0625 |
Table S14. ANOVA and Tukey’s test tables for Valeric acid production in YH1-B1 mono- and co-cultures

## Slide 16
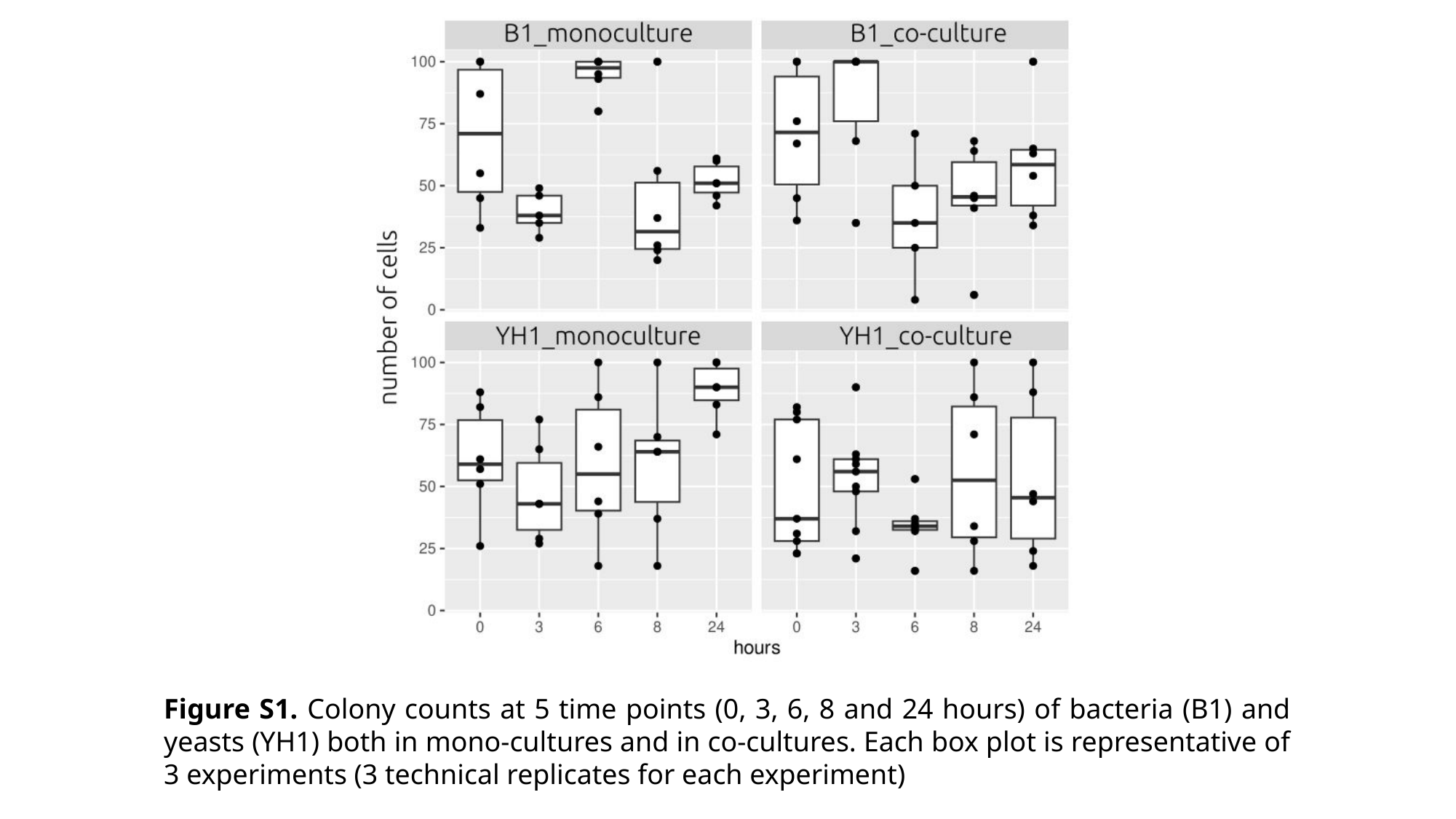

Figure S1. Colony counts at 5 time points (0, 3, 6, 8 and 24 hours) of bacteria (B1) and yeasts (YH1) both in mono-cultures and in co-cultures. Each box plot is representative of 3 experiments (3 technical replicates for each experiment)

## Slide 17
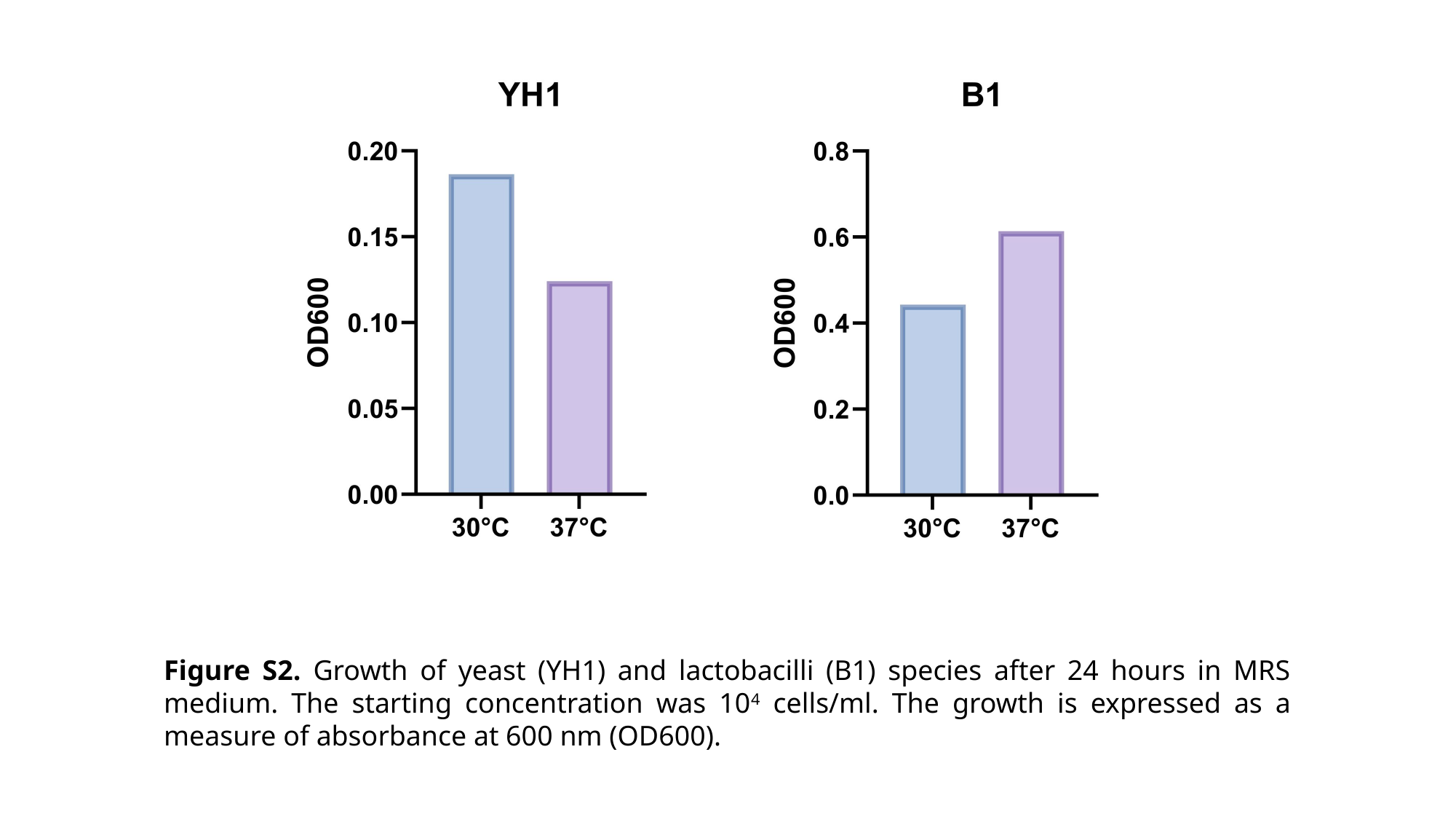

Figure S2. Growth of yeast (YH1) and lactobacilli (B1) species after 24 hours in MRS medium. The starting concentration was 104 cells/ml. The growth is expressed as a measure of absorbance at 600 nm (OD600).

## Slide 18
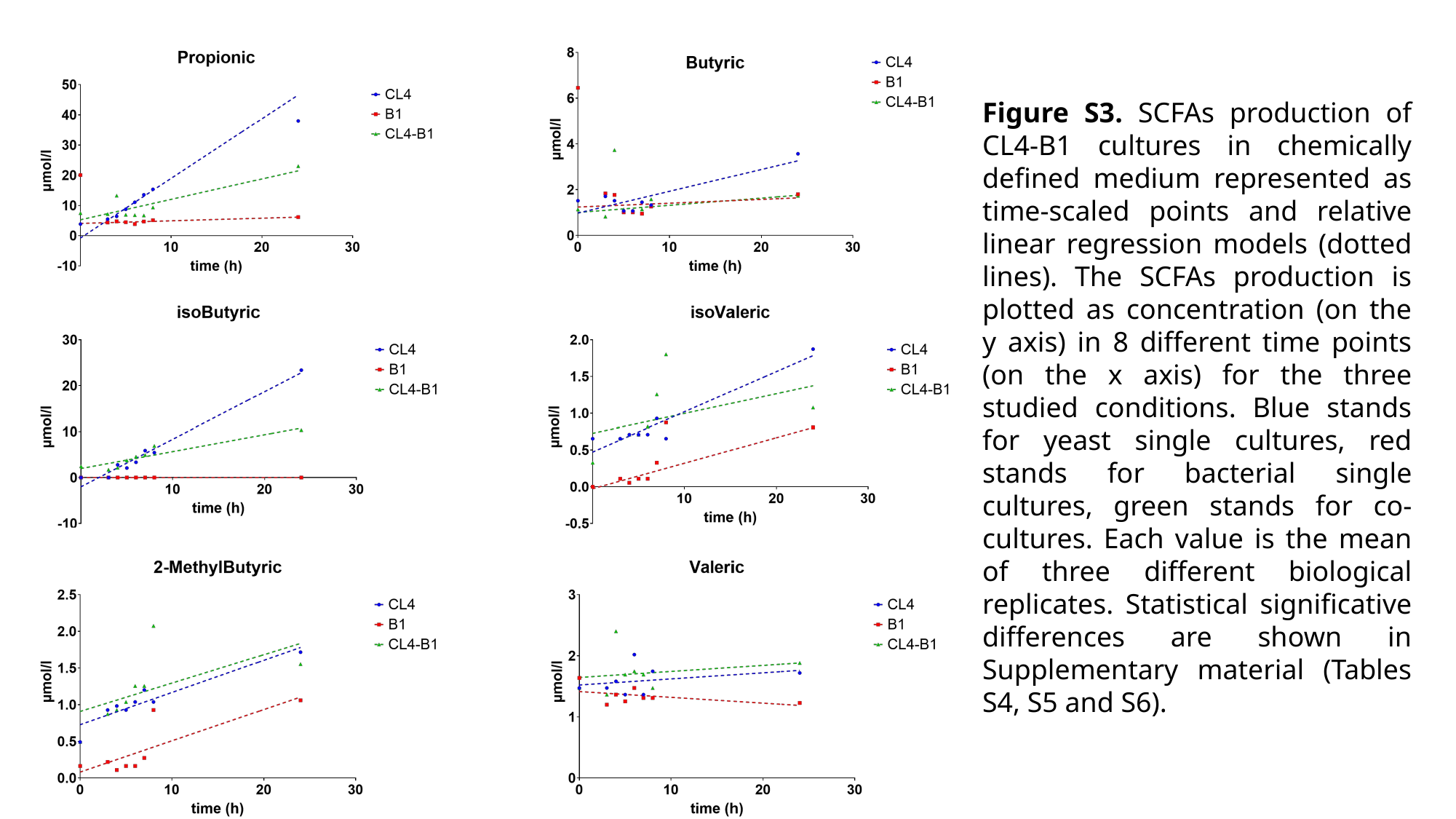

Figure S3. SCFAs production of CL4-B1 cultures in chemically defined medium represented as time-scaled points and relative linear regression models (dotted lines). The SCFAs production is plotted as concentration (on the y axis) in 8 different time points (on the x axis) for the three studied conditions. Blue stands for yeast single cultures, red stands for bacterial single cultures, green stands for co-cultures. Each value is the mean of three different biological replicates. Statistical significative differences are shown in Supplementary material (Tables S4, S5 and S6).

## Slide 19
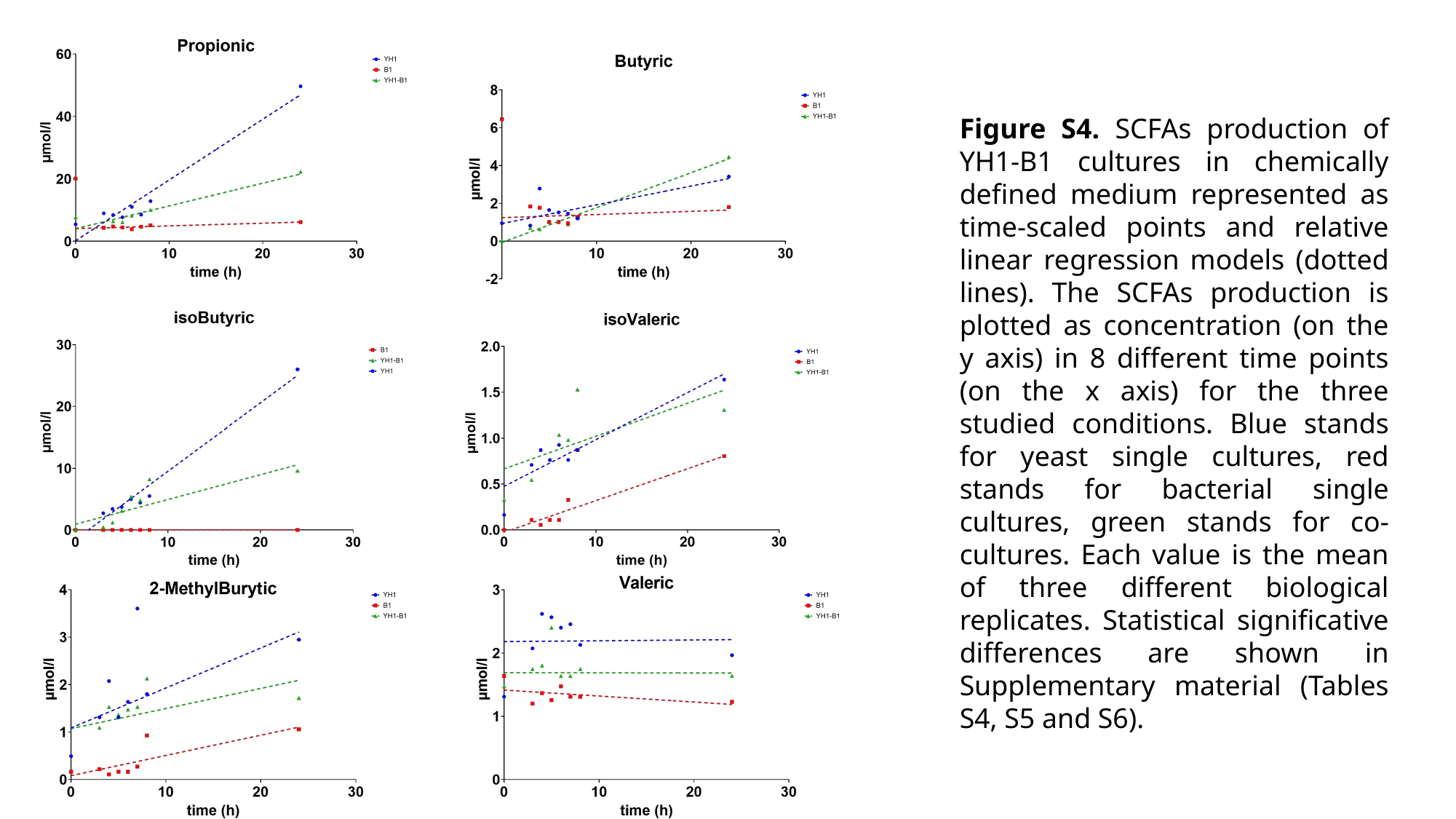

Figure S4. SCFAs production of YH1-B1 cultures in chemically defined medium represented as time-scaled points and relative linear regression models (dotted lines). The SCFAs production is plotted as concentration (on the y axis) in 8 different time points (on the x axis) for the three studied conditions. Blue stands for yeast single cultures, red stands for bacterial single cultures, green stands for co-cultures. Each value is the mean of three different biological replicates. Statistical significative differences are shown in Supplementary material (Tables S4, S5 and S6).

## Slide 20
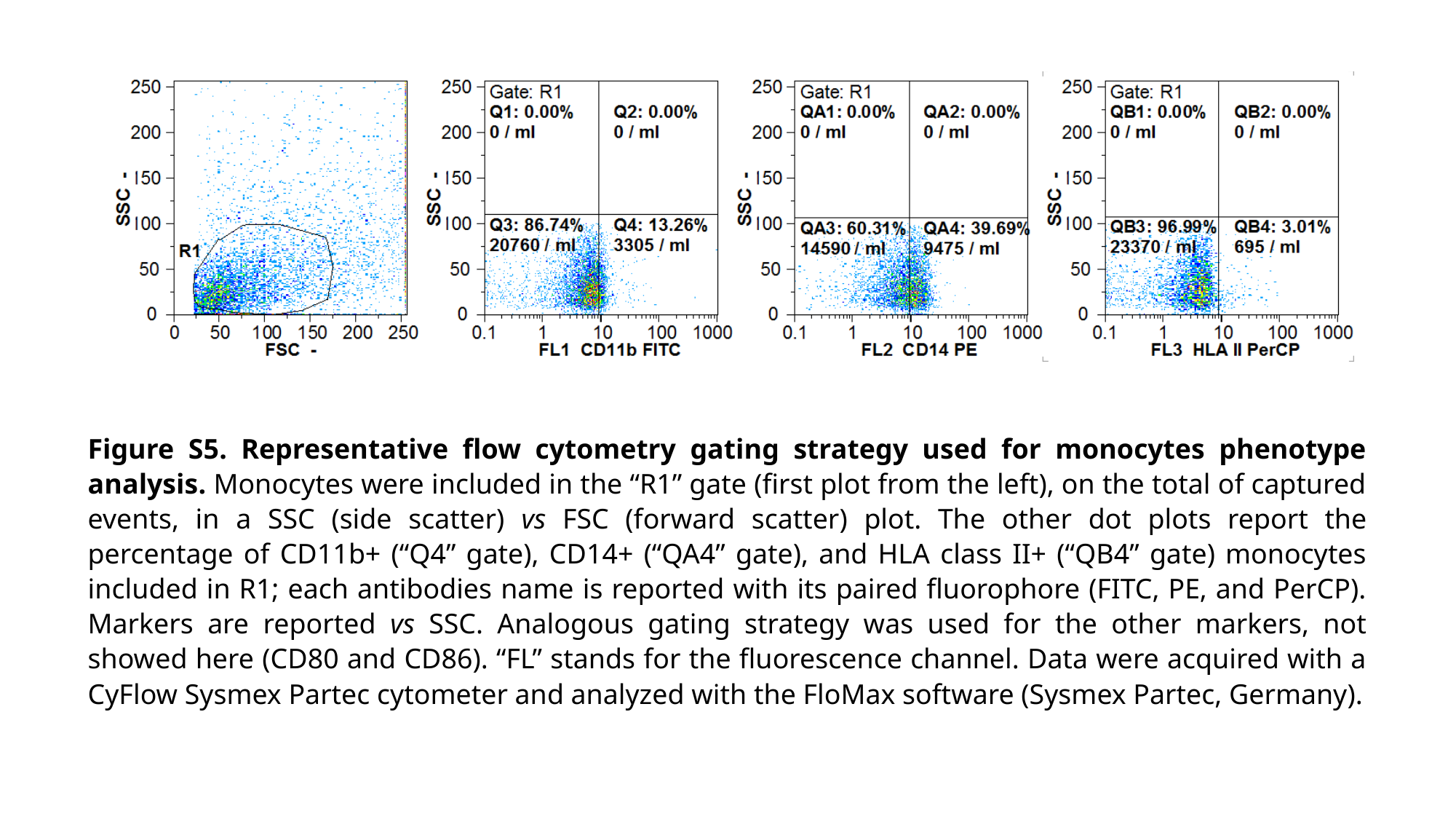

Figure S5. Representative flow cytometry gating strategy used for monocytes phenotype analysis. Monocytes were included in the “R1” gate (first plot from the left), on the total of captured events, in a SSC (side scatter) vs FSC (forward scatter) plot. The other dot plots report the percentage of CD11b+ (“Q4” gate), CD14+ (“QA4” gate), and HLA class II+ (“QB4” gate) monocytes included in R1; each antibodies name is reported with its paired fluorophore (FITC, PE, and PerCP). Markers are reported vs SSC. Analogous gating strategy was used for the other markers, not showed here (CD80 and CD86). “FL” stands for the fluorescence channel. Data were acquired with a CyFlow Sysmex Partec cytometer and analyzed with the FloMax software (Sysmex Partec, Germany).

## Slide 21
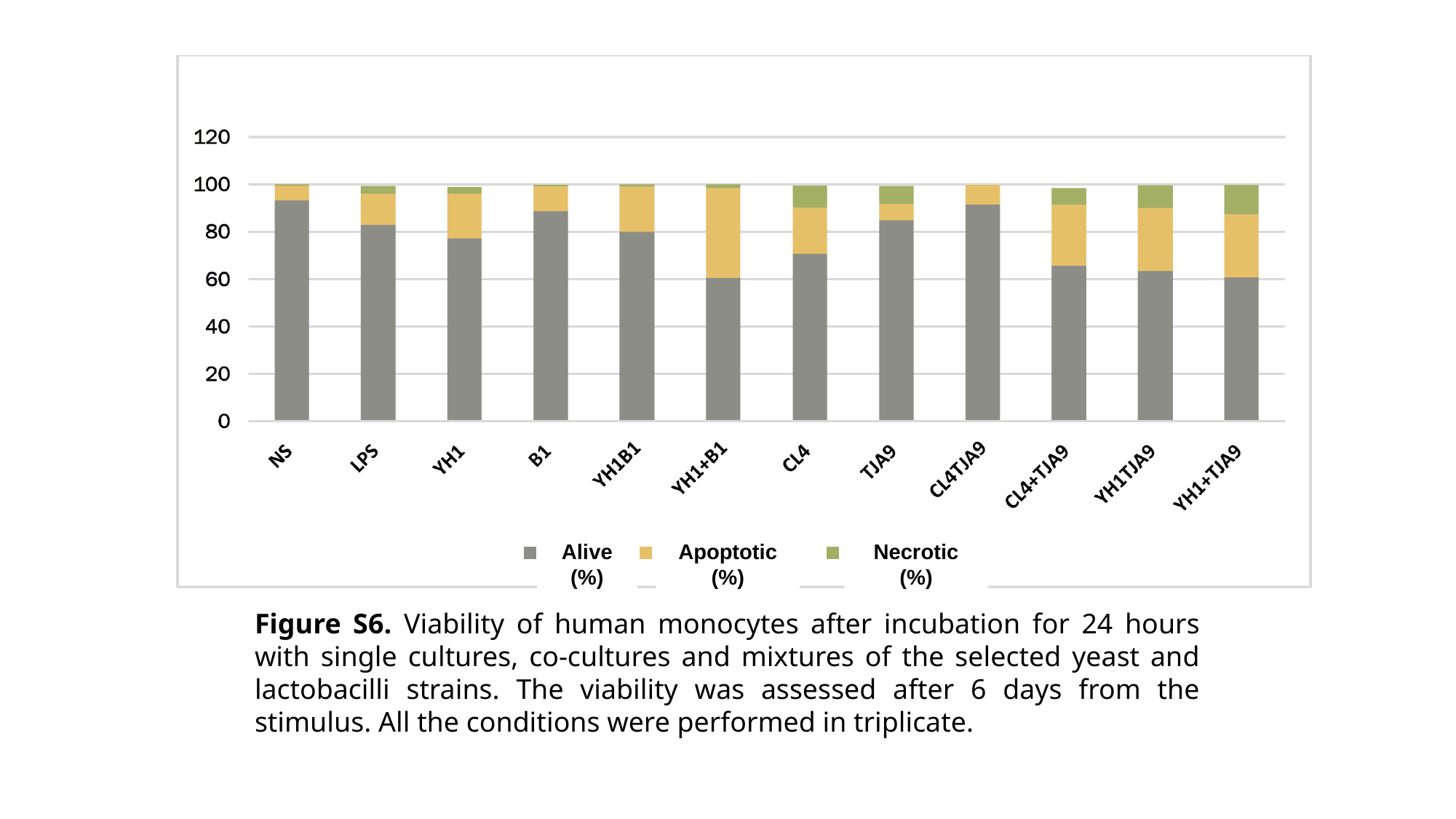

Necrotic (%)
Apoptotic (%)
Alive (%)
Necrotic (%)
Apoptotic (%)
Alive (%)
Figure S6. Viability of human monocytes after incubation for 24 hours with single cultures, co-cultures and mixtures of the selected yeast and lactobacilli strains. The viability was assessed after 6 days from the stimulus. All the conditions were performed in triplicate.

## Slide 22
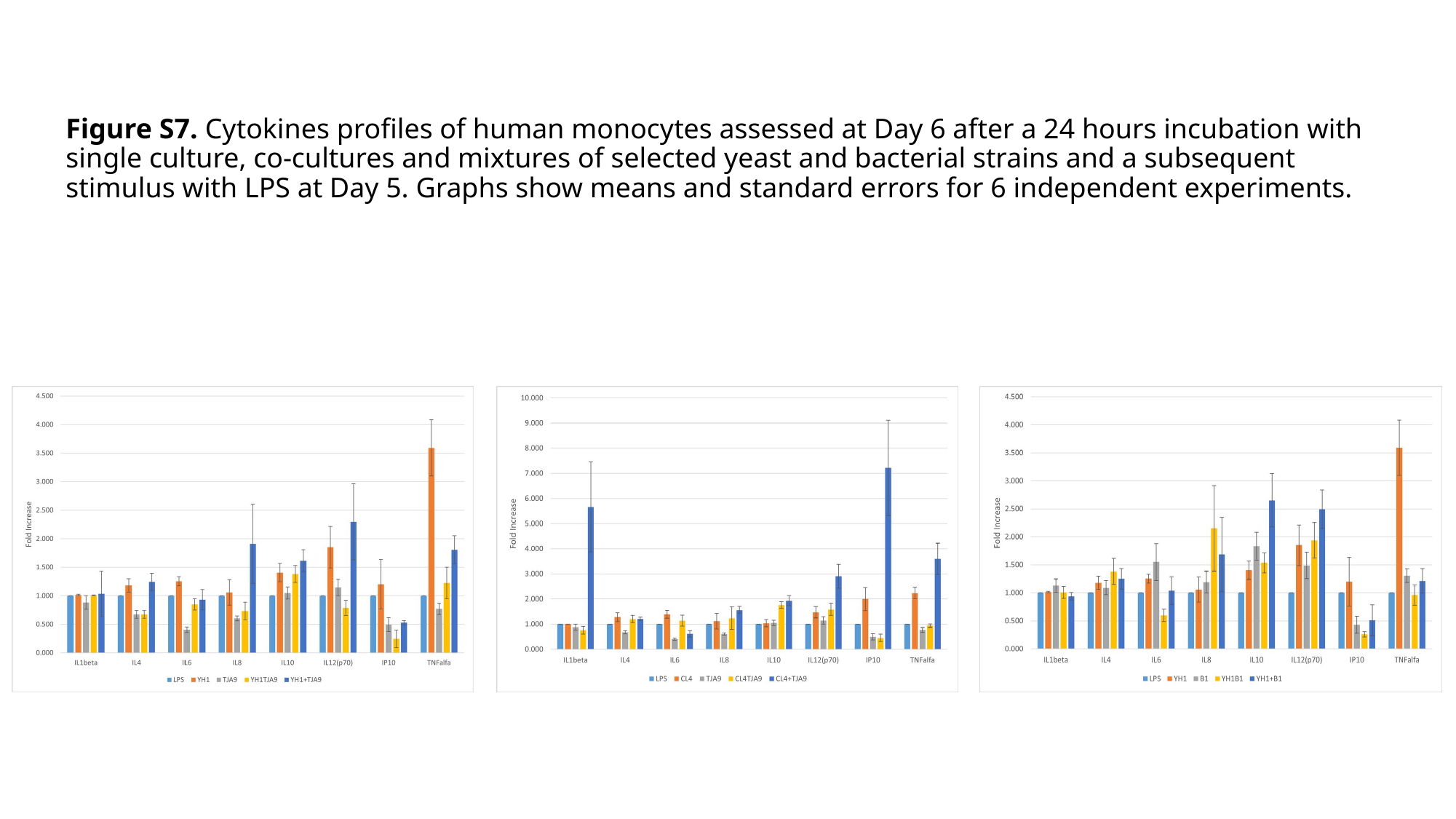

Figure S7. Cytokines profiles of human monocytes assessed at Day 6 after a 24 hours incubation with single culture, co-cultures and mixtures of selected yeast and bacterial strains and a subsequent stimulus with LPS at Day 5. Graphs show means and standard errors for 6 independent experiments.
